# The androgen receptor controls expression of the cancer-associated sTn antigen and cell adhesion through induction of ST6GalNAc1 in prostate cancer

**DOI:** 10.18632/oncotarget.6024

**Published:** 2015-10-07

**Authors:** Jennifer Munkley, Sebastian Oltean, Daniel Vodák, Brian T. Wilson, Karen E. Livermore, Yan Zhou, Eleanor Star, Vasileios I. Floros, Bjarne Johannessen, Bridget Knight, Paul McCullagh, John McGrath, Malcolm Crundwell, Rolf I. Skotheim, Craig N. Robson, Hing Y. Leung, Lorna W. Harries, Prabhakar Rajan, Ian G. Mills, David J. Elliott

**Affiliations:** ^1^ Institute of Genetic Medicine, Newcastle University, Newcastle-upon-Tyne, UK; ^2^ Microvascular Research Laboratories, School of Physiology and Pharmacology, University of Bristol, Bristol, UK; ^3^ Bioinformatics Core Facility, Institute for Cancer Genetics and Informatics, The Norwegian Radium Hospital, Oslo University Hospital, Oslo, Norway; ^4^ Northern Genetics Service, Newcastle Upon Tyne NHS Foundation Trust, Newcastle-upon-Tyne, UK; ^5^ Beatson Institute for Cancer Research, Glasgow, UK; ^6^ Department of Molecular Oncology, Institute for Cancer Research, Oslo University Hospital, Oslo, Norway; ^7^ NIHR Exeter Clinical Research Facility, Royal Devon and Exeter NHS Foundation Trust, Exeter, UK; ^8^ Department of Pathology, Royal Devon and Exeter NHS Foundation Trust, Exeter, UK; ^9^ Exeter Surgical Health Services Research Unit, Royal Devon and Exeter NHS Foundation Trust, Exeter, UK; ^10^ Department of Urology, Royal Devon and Exeter NHS Foundation Trust, Exeter, UK; ^11^ Northern Institute for Cancer Research, Newcastle University, Newcastle-upon-Tyne, UK; ^12^ Institute of Cancer Sciences, University of Glasgow, Glasgow, UK; ^13^ Institute of Biomedical and Clinical Sciences, University of Exeter, Devon, UK; ^14^ Prostate Cancer Research Group, Centre for Molecular Medicine Norway (NCMM), University of Oslo and Oslo University Hospitals, Oslo, Norway; ^15^ Departments of Molecular Oncology, Institute of Cancer Research and Radium Hospital, Oslo, Norway; ^16^ PCUK/Movember Centre of Excellence for Prostate Cancer Research, Centre for Cancer Research and Cell Biology (CCRCB), Queen's University, Belfast, UK

**Keywords:** androgens, prostate cancer, ST6GalNAc1, glycosylation, Sialyl-Tn (sTn)

## Abstract

Patterns of glycosylation are important in cancer, but the molecular mechanisms that drive changes are often poorly understood. The androgen receptor drives prostate cancer (PCa) development and progression to lethal metastatic castration-resistant disease. Here we used RNA-Seq coupled with bioinformatic analyses of androgen-receptor (AR) binding sites and clinical PCa expression array data to identify *ST6GalNAc1* as a direct and rapidly activated target gene of the AR in PCa cells. *ST6GalNAc1* encodes a sialytransferase that catalyses formation of the cancer-associated sialyl-Tn antigen (sTn), which we find is also induced by androgen exposure. Androgens induce expression of a novel splice variant of the ST6GalNAc1 protein in PCa cells. This splice variant encodes a shorter protein isoform that is still fully functional as a sialyltransferase and able to induce expression of the sTn-antigen. Surprisingly, given its high expression in tumours, stable expression of ST6GalNAc1 in PCa cells reduced formation of stable tumours in mice, reduced cell adhesion and induced a switch towards a more mesenchymal-like cell phenotype *in vitro*. *ST6GalNAc1* has a dynamic expression pattern in clinical datasets, being significantly up-regulated in primary prostate carcinoma but relatively down-regulated in established metastatic tissue. ST6GalNAc1 is frequently upregulated concurrently with another important glycosylation enzyme GCNT1 previously associated with prostate cancer progression and implicated in Sialyl Lewis X antigen synthesis. Together our data establishes an androgen-dependent mechanism for sTn antigen expression in PCa, and are consistent with a general role for the androgen receptor in driving important coordinate changes to the glycoproteome during PCa progression.

## INTRODUCTION

Prostate cancer (PCa) development and progression is driven by the androgen receptor (AR). Androgen deprivation therapy (ADT) is the first line of treatment for PCa, and although it is usually initially effective, the recurrence of castrate resistant PCa (CRPCa) is common and ultimately lethal. Progression to CRPCa is thought to involve persistence of AR signalling and reprogramming of the AR transcriptional landscape [1, 2]. Androgen-regulated genes have been identified by genome/transcriptome-wide analyses of AR binding sites and downstream changes in gene expression in response to androgen exposure. The identified genes have included regulators of cell cycle, biosynthetic and glycolysis pathways, and the master regulators thereof, such as calcium/calmodulin-dependent protein kinase 2 (*CAMKK2*) [3].

Given the importance of metastasis in PCa mortality, a key objective is to identify mechanisms that contribute to cell adhesion and migration. Changes in cell surface glycosylation are commonly observed during malignancy with frequent over-expression of sialylated antigens [4-6]. A well-known cancer-associated glycan structure is a short O-glycan containing a sialic acid residue known as sialyl-Tn (sTn) antigen. sTn is synthesised by the enzyme ST6GalNAc1 which catalyses transfer of a sialic acid in ɑ2-6 linkage onto the Tn antigen (GalNAca1-OSer/Thr), and modifies the glycosylation pattern of various membrane O-glycoproteins [7]. The cellular expression status of glycan structures is critical for cell adhesion, invasion and metastasis. In PCa the glyco-phenotype of transformed cells often becomes modified with a sharp rise in sialylation of O-glycans [8].

Although patterns of glycosylation change in cancer, the underlying mechanisms driving these changes are not well understood. Here, we use RNA-Seq in the androgen sensitive LNCaP PCa cell line [9], coupled to genome-wide mapping data for AR binding sites [3] and clinical PCa expression array data [10] to identify an androgen-driven mechanism of glycosylation change. This mechanism involves a new splice variant of ST6GalNAc1 which encodes a novel short protein isoform that controls PCa cell adhesion.

## RESULTS

### *ST6GalNAc1* is upregulated in primary prostate cancer and is an early and direct target of the AR

We used RNA-Seq to monitor androgen-mediated changes in the transcriptome of LNCaP cells treated with 10 nM of the synthetic androgen analogue R1881 (methyltrienolone) for 24 hours. The Cufflinks package reported 674 up- and 1834 down- regulated genes (*p* < 0.0075, Supplementary Figure 1 and Supplementary Tables 2 and 3). The RNA-Seq reads aligned to the human genome (hg19) can be visualised for any gene using the following link: http://genome-euro.ucsc.edu/cgi-bin/hgTracks?db=hg19&position=chr17%3A74620838-74639920&hgsid=208866799_IfRA3VMoSbPBVAhT3NJysAg6KahE

**Figure 1:**
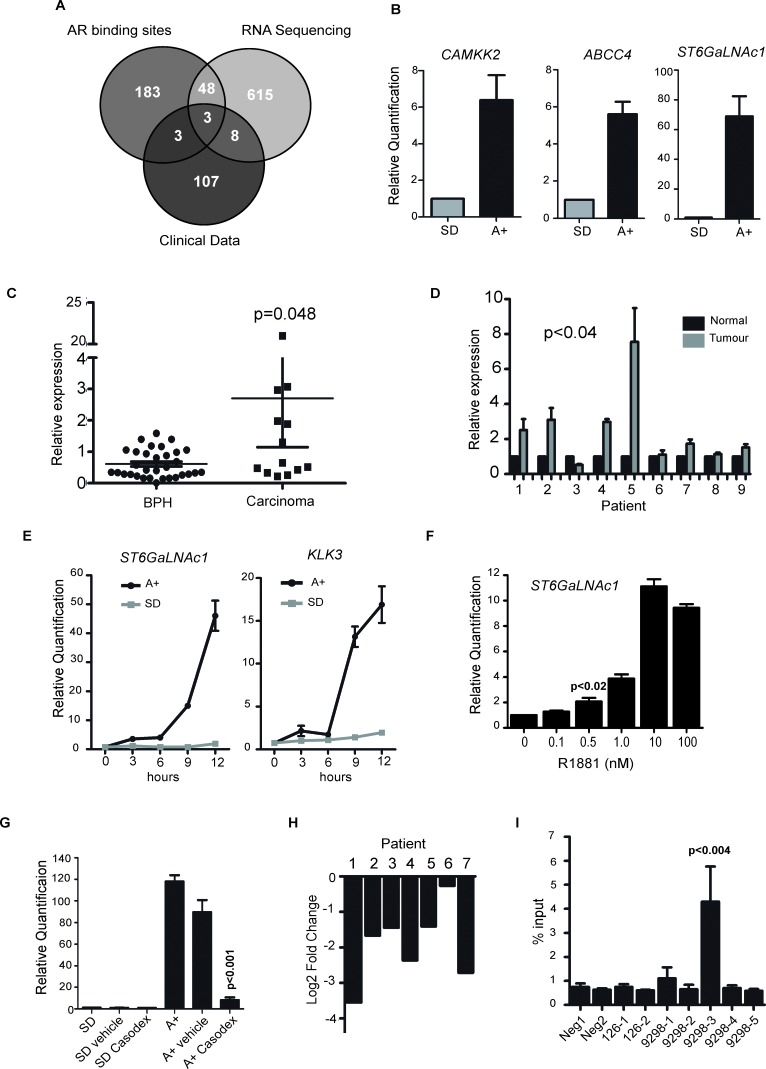
ST6GalNAc1 is an early and direct target of the AR and is upregulated in primary prostate tumours **A.** RNA-sequencing was carried out on RNA extracted from LNCaP cells grown in media supplemented with 10% charcoal dextran stripped FBS (steroid deplete media, SD) or stimulated with 10nM synthetic androgen analogue methyltrienolone (R1881) for 24 hours (androgens, A+). We compared genes up-regulated in response to R1881 with published AR binding sites [3] and clinical PCa expression array data [10] (GSE35988, downloaded from the NCBI GEO data repository). The criteria applied were the presence of an AR binding site within 50kb of the transcription start site of the gene, significant differential gene expression in the clinical dataset [10] (genes over-expressed by 1.6 fold or more in cancer vs. normal when profiled using an array platform (Agilent-012391 Whole Human Genome Oligo Microarray G4112A)), and evidence of androgen-regulated expression in the RNA-Seq data (fold change > 1.6 as reported by the cufflinks package). This identified three genes which overlapped between all three data sets, *CAMKK2*, *ABCC4* and *ST6GalNAc1*. **B.** We confirmed androgen-regulation of these genes by real-time PCR (using 3 independent sample sets treated with 10nM R1881 for 24 hours) and identified *ST6GalNAc1* as *a* novel androgen regulated gene. **C.** Real-time PCR analysis of *ST6GalNAc1* mRNA from 32 benign samples from patients with benign prostatic hyperplasia (BPH) and 17 malignant samples from transurethral resection of the prostate (TURP) samples. **D.** We also analysed RNA from normal and matched PCa tissue from 9 patients obtained by radical prostectomy. **E.** Real-time PCR analysis of *ST6GalNAc1* mRNA in LNCaP cells stimulated with 10 nM R1881 (A+) or without (SD) over 24 hours, showed increased expression after less than 3 hours of androgen stimulation; a similar pattern is seen for KLK3 (PSA). **F.** Induction of *ST6GalNAc1* is also evident in LNCaP cells treated with 0.5 to 100nM of R1881 for 24 hours. Relative *ST6GalNAc1* expression was detected by real-time PCR. **G.** Up-regulation of *ST6GalNAc1* mRNA in response to treatment with 10 nM R1881 for 24 hours is negated by the AR antagonist 10μM Casodex^®^ (bicalutamide) but not by ethanol alone (vehicle). **H.** Analysis of publically available patient RNA-seq data pre- and post- androgen ablation therapy for *ST6GalNAc1* shows that following androgen ablation there is a down-regulation of *ST6GalNAc1* mRNA for all 7 patients tested[63]. **I.** AR-ChIP in LNCaP cells treated with 10nM R1881 for 24 hours revealed one AR binding sites in close proximity to the *ST6GalNAc1* gene (for details of the binding sites see Supplementary Figure 2).

A comparison of RNA-Seq data with our previously published exon microarray data [11] showed an 86% overlap, with > 4 times more differentially expressed genes identified by RNA-Seq (Supplementary Table 4). To identify genes of potential clinical interest we compared genes up-regulated in response to R1881 with published AR binding sites [3] and clinical PCa expression array data [10] (GSE35988, downloaded from the NCBI GEO data repository). The criteria applied were the presence of an AR binding site within 50kb of the transcription start site of the gene, significant differential gene expression reported in the clinical dataset [10] and evidence of androgen-regulated expression in the LNCaP RNA-Seq data. Three genes fulfilled these stringent selection criteria (Figure 1A and Supplementary Tables 5 and 6). Two of these three overlapping genes already have established roles in clinical PCa. These were *CAMKK2* which is an important determinant of central metabolism and is over-expressed in PCa [3]; and the ATP-binding cassette transporter *ABCC4* that is implicated in disease progression and resistance of PCa cells to nucleotide-based chemotherapeutic drugs [12]. Also found within this overlapping subgroup was the *ST6GalNAc1* gene. Both *CAMKK2* and *ABCC4* are previously known to be activated in response to androgens, and using qRT-PCR we similarly confirmed strong androgen-dependent induction of these *ST6GalNAc1* genes (Figure 1B).

To examine if expression of the *ST6GalNAc1* gene becomes changed in clinical prostate cancer we carried out meta-analysis of 544 prostate tumours using data from 7 previously published studies [10, 13-17]. We found that 6/7 datasets showed significant up-regulation of *ST6GalNAc1* mRNA expression in prostate carcinoma versus normal prostate tissue (Supplementary Table 7). *ST6GalNAc1* expression showed an average mean fold change of 2.951 (*p* = 5.18E-7) in 122 primary PCa samples studied by Grasso et al. [10], and a mean fold change of 4.049 (*p* = 1.99E-6) in samples studied by Varambally et al. [17], with *ST6GalNAc1* ranked in the top 1% of over-expressed genes. In order to test the results of this meta-analysis, we further analysed *ST6GalNAc1* expression in a panel of clinical PCa samples by qRT-PCR. We found *ST6GalNAc1* mRNA was significantly up-regulated in prostate carcinoma relative to BPH tissue (*p* = 0.048), and in primary prostate tumour tissue relative to matched normal tissue from the same patient (*p* < 0.04) (Figure 1D).

Induction of *ST6GalNAc1* gene expression was extremely rapid and could be detected < 3 hours after androgen exposure suggesting it is directly regulated by the AR. The early expression profile of *ST6GalNAc1* following androgen exposure had similar dynamics to the known directly AR-regulated gene *KLK3* (Figure 1E). Androgen-mediated induction of *ST6GalNAc1* expression was induced over a range of R1881 concentrations consistent with *ST6GalNAc1* induction also occurring under physiological conditions within the prostate (Figure 1F), and blocked by treatment with the AR antagonist Casodex^®^ (bicalutamide) (Figure 1G). Analysis of previously published RNA-Sequencing data from 7 PCa patients [63] showed that *ST6GalNAc1* gene expression is also strongly down-regulated following ADT in all cases (Figure 1H), suggesting that *ST6GalNAc1* is also regulated (either directly or indirectly) by androgens *in vivo*, and is clinically relevant in patients.

Multiple AR binding sites (ARBSs) situated both upstream and within the *ST6GalNAc1* gene had been previously predicted by ChIP-Seq [3]. We confirmed direct AR binding in LNCaP cells at an ARBS in close proximity to *ST6GalNAc1* using ChIP-qPCR (*p* **<** 0.004) (Figure 1I and Supplementary Figure 2). Induction of *ST6GalNAc1* mRNA expression in response to androgens was further confirmed in the androgen-responsive VCaP cell line (Supplementary Figure 2). In VCaP cells, which have amplification of the AR [18], we found six sites of AR binding within 50Kb of the *ST6GalNAc1* gene (Supplementary Figure 2). Androgen induction of *ST6GalNAc1* was very specific: no parallel induction of the *ST6GalNac2-6* genes was observed in response to androgen exposure (Supplementary Figure 3).

### A novel isoform of *ST6GalNAc1* is expressed in prostate cells in response to androgen stimulation

**Figure 2:**
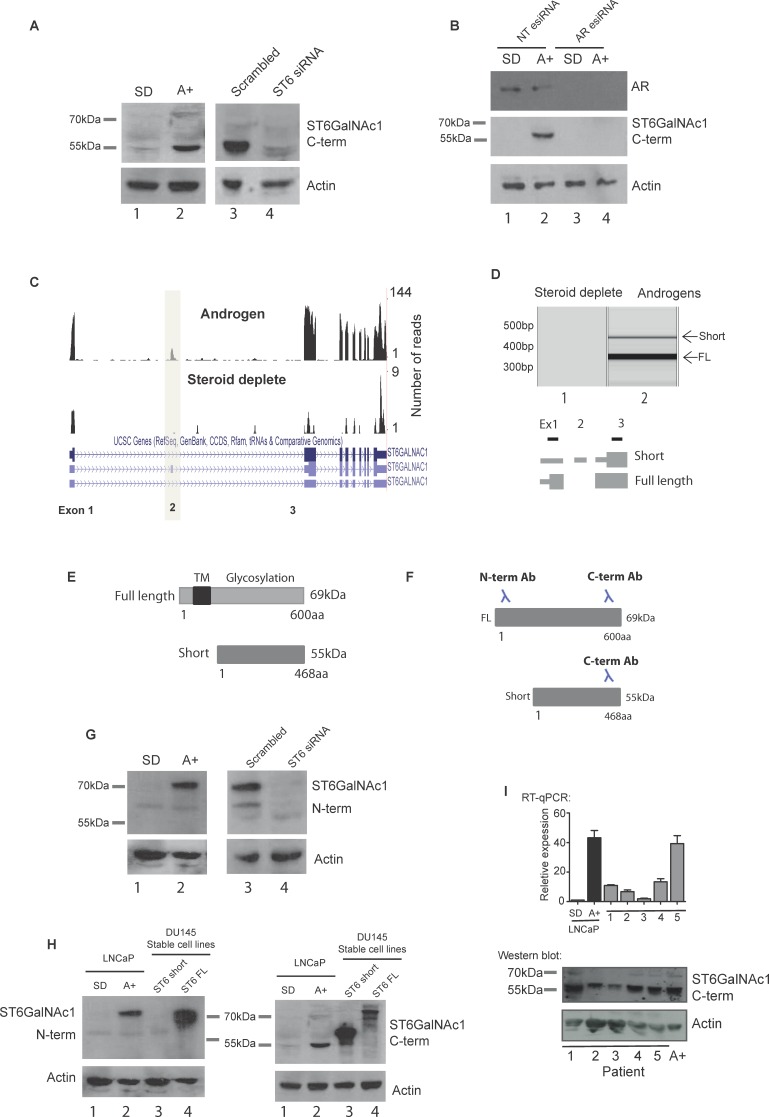
A novel alternative splice isoform of ST6GalNAc1 is expressed in response to androgen stimulation **A.** Androgen regulation of ST6GalNAc1 in LNCaP cells was confirmed at the protein level by western blot (A, left panels). This revealed a band at approximately 55KDa which was not detected in cells grown in the absence of hormone (steroid deplete, SD), but induced by treatment with 10 nM of the synthetic androgen R1881 for 24 hours (A+). Depletion of *ST6GalNAc1* in androgen stimulated cells using siRNA (A, right panels) confirmed the specificity of the band. **B.** Depletion of AR protein in LNCaP cells by esiRNA shows that when the AR is depleted there is no induction of ST6GalNAc1 protein in response to androgens (cells were treated with or without 10nM R1881 for 48 hours). **C.** Visualisation of our LNCaP cell RNA-seq reads (see Figure 1 for details) on the UCSC genome browser identified a novel isoform of *ST6GalNAc1* which includes an additional exon (exon 2). This isoform has a longer 5′ untranslated region and is predicted to produce a shorter 55kDa protein which is missing the first 132 amino acids. **D.** PCR using primers specific to exon 1 and 3 produced two bands indicating the presence of two *ST6GalNAc1* mRNA isoforms (with and without exon 2) in LNCaP cells treated with 10nM R1881 for 24 hours. **E.** Model showing predicted localisation domains in ST6GalNAc1. ST6GalNAc1-short is missing a predicted transmembrane localisation domain from amino acids 15-35 (http://www.uniprot.org/uniprot/Q9NSC7). **F.** The ST6GalNAc1 antibody used in **A.** was raised against the C-terminal of the protein, meaning that it will detect both isoforms. An additional antibody to the N-terminal of ST6GalNAc1 will not detect the 55kDa ST6GalNAc1-short protein. **G.** Detection of ST6GalNAc1 using the N-terminal antibody confirms that the 69kDa ST6GalNAc1 protein is also expressed, although at presumably at much lower level that the 55kDa isoform. Depletion of *ST6GalNAc1* using siRNA confirmed the specificity of the band (G, right panels). **H.** Western blot detection of FLAG-tagged over-expressed ST6GalNAc1 and ST6GalNAc1-short by the N- and C-terminal antibodies in DU145 cell lines. As predicted the N-terminal antibody does not detect ST6GalNAc1-short but does detect full length protein (H, left panel, lanes 3 and 4 respectively). The C-terminal antibody detects both isoforms (H, right panel, lanes 3 and 4 respectively). **I.** ST6GalNAc1-short is detected in a panel of 5 PCa samples by real-time PCR (using primers specific to exon 2), and at the protein level by western blot. LNCaP cells grown in the presence of 10 nM of the synthetic androgen R1881 for 48 hours were used as a positive control.

The above data indicated that *ST6GalNAc1* gene expression is induced by the AR in LNCaP and VCaP cells, and reciprocally repressed by ADT in patients with PCa. We thus tested if *ST6GalNAc1* protein is also induced by androgens. Surprisingly, rather than the previously described 69KDa full length *ST6GalNAc1* canonical protein isoform, we detected predominant androgen-inducible expression of a ∼55KDa protein in LNCaP cells on western blots, using an antibody specific to the C-terminal of the *ST6GalNAc1* protein (Figure 2A). Expression of this ∼55KDa protein isoform was sensitive to siRNA-mediated depletion of *ST6GalNAc1* (Figure 2A), and was not induced in androgen treated LNCaP cells depleted of the AR (Figure 2B), indicating it is a novel androgen regulated isoform of ST6GalNAc1 protein.

Further investigation indicated this novel 55 kDa protein isoform is encoded by a novel splice isoform. The canonical 69KDa ST6GalNAc1 protein isoform is encoded by a splice isoform of mRNA which skips exon 2 (NCBI Reference Sequence: NM_018414.4). Closer inspection of our RNA-Seq data showed reads mapping to exon 2 of *ST6GalNAc1* following androgen treatment, indicating that exon 2 is also specifically included in LNCaP cells in response to androgens as well as induction of the entire gene (Figure 2C and Supplementary Figure 4). Inclusion of *ST6GalNAc1* exon 2 leads to an mRNA with a longer 5′ UTR, that uses an alternative downstream translational start codon predicted to encode a 55KDa protein (NCBI Reference Sequence: NM_001289107.1). In androgen-treated LNCaP cells we detected *ST6GalNAc1* mRNA both with and without exon 2 using RT-PCR (Figure 2D). We also detected *ST6GalNAc1* exon 2 inclusion in a number of other PCa cell lines by RT-PCR (Supplementary Figure 5A, 5B).

The novel 55kDa protein isoform of ST6GalNAc1 is missing 132 amino acids from the N-terminus, including a predicted trans-membrane domain (Figure 2E) and an epitope recognised by another α-N-terminal ST6GalNAc1 antibody (Figure 2F). Using western blots and this α-N-terminal ST6GalNAc1 antisera, we detected the canonical 69KDa ST6GalNAc1 protein in LNCaP cells, and found that this was also androgen-inducible and sensitive to siRNA-mediated depletion of *ST6GalNAc1* (Figure 2G). Taken together these data show the 69KDa ST6GalNAc1 protein isoform is also produced in LNCaP cells, but at lower levels than the shorter 55KDa isoform, and both are androgen inducible, which would be expected since they are transcribed from the same promoter. We further confirmed the relative sizes of the two ST6GalNAc1 protein isoforms through detection of over-expressed protein with both antibodies (Figure 2H: note these over-expressed proteins in DU145 cells are fusion proteins, so migrate slightly slower than their endogenous counterpart proteins).

Analysis of publically available RNA-Seq data [19] show that *ST6GalNAc1* exon 2 is detected in both clinical prostate cancer and in a number of additional cancers, particularly lung cancer (Supplementary Figure 5C). Interrogation of the Taylor et al. dataset [16] revealed that in clinical PCa samples *ST6GalNAc1* exon 2 is expressed at similar levels to other exons in the *ST6GalNAc1* gene (Supplementary Figure 6), suggesting that it is also the short isoform of *ST6GalNAc1* that is expressed in PCa tumours. Confirming that this short protein is the major ST6GalNAc1 protein isoform expressed in clinical prostate cancer, the 55KDa ST6GalNAc1 protein isoform was also detected in androgen-treated VCaP cells (Supplementary Figure 2), and in clinical prostate samples by western blot analysis in 5 out of 5 tissue lysates (Figure 2I).

### Androgen exposure induces expression of the sTn antigen in PCa cells

**Figure 3:**
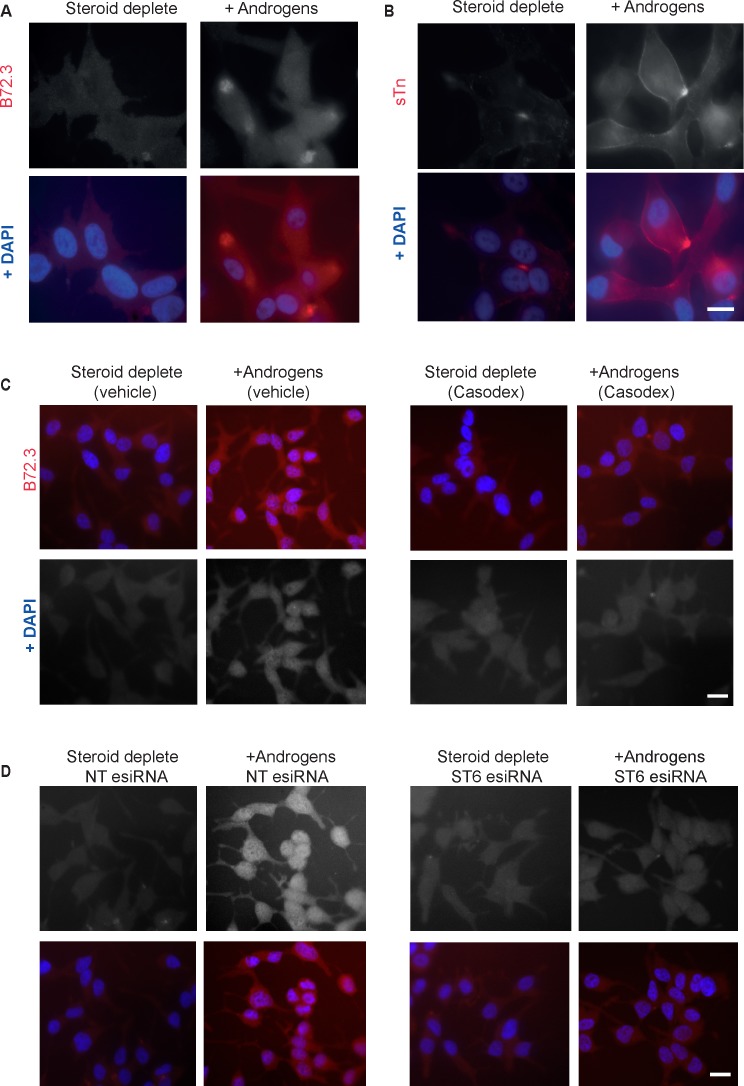
The sialyl-Tn antigen is androgen-regulated in LNCaP cells Analysis of glycosylation in LNCaP cells grown in the presence or absence of androgens (10nM R1881) for 72 hours by immunofluorescence. Androgen-mediated up-regulation of sTn antigen (formed by ST6GalNAc1 catalysed sialylation of GalNAc residues) is observed using two different antibodies **A.**, **B.** Induction of sTn by androgens is inhibited by the presence of 10μM Casodex^®^ (bicalutamide) **C.** and by esiRNA mediated depletion of ST6GalNAc1 **D.**. Bar is 10 μm.

Given the strong induction of *ST6GalNAc1* in response to androgen exposure, we tested if the sTn antigen might also be androgen-regulated. Consistent with this prediction, two independent monoclonal antibodies specific to sTn antigen detected androgen-activated expression of this cancer-associated antigen in LNCaP cells (Figure 3A, 3B). Induction of the sTn antigen by androgens in LNCaP cells was inhibited by the AR antagonist Casodex^®^ (bicalutamide) (Figure 3C), by esiRNA mediated depletion of ST6GalNAc1 (Figure 3D), and by esiRNA mediated depletion of the AR (Supplementary Figure 7) indicating a direct mechanism of induction. Induction of sTn antigen by androgen treatment was also evident in VCaP cells (Supplementary Figure 2).

### The short isoform of ST6GalNAc1 functions as a sialyltransferase in PCa cells and induces sTn

**Figure 4:**
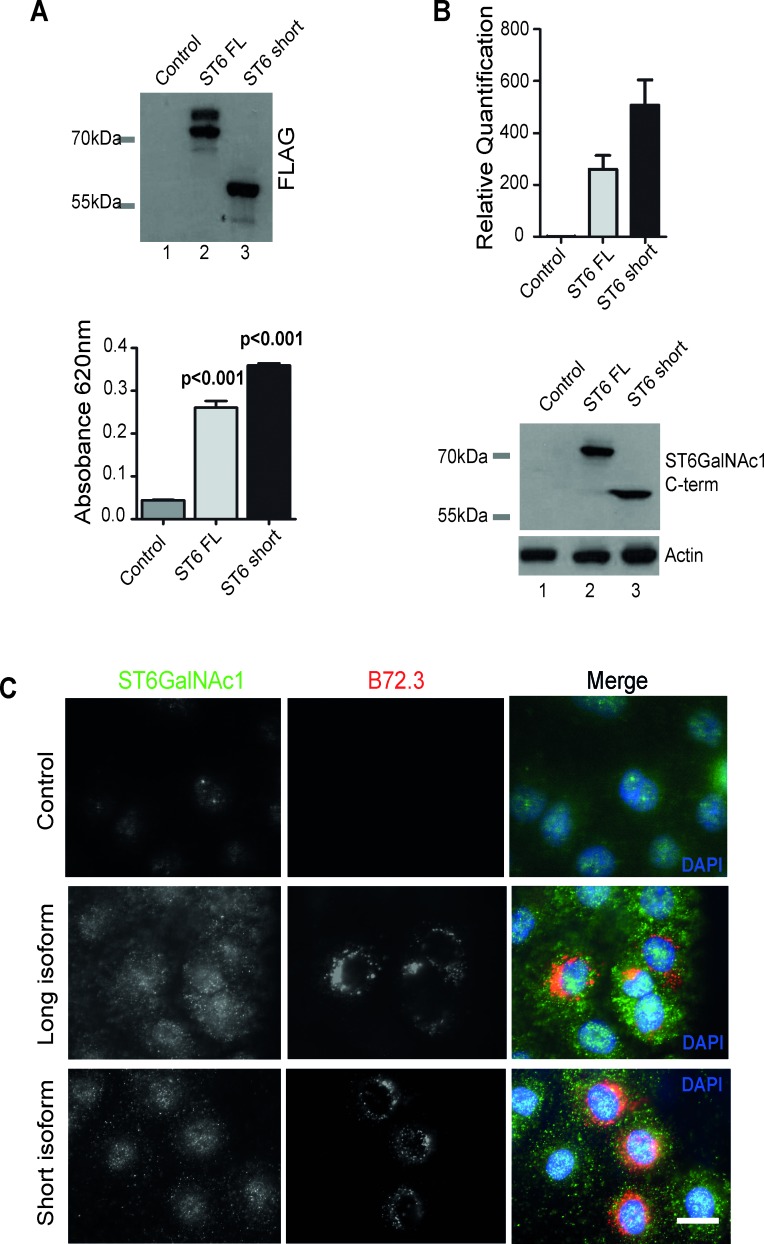
The short isoform of ST6GalNAc1 functions as a sialyltransferase in PCa cells and induces expression of sTn **A.** FLAG-tagged long and short isoforms of ST6GalNacI were purified from Flp-In HEK293 cells by immunoprecipitation. Isolation was confirmed by western blot (A, upper panel). The purified proteins were then used directly in an *in vitro* sialylation assay (A, lower panel). Phosphate standard (R&D Systems EA002) was used as a positive control and to produce a standard curve. Despite missing the first 132 amino acids *ST6GalNAc1-short* is still able to function as a sialyltransferase *in vitro*. **B.** Detection of *ST6GalNAc1* by real-time PCR and western blot in DU145 cells stably transfected with either empty vector (lane 1), full length FLAG-tagged *ST6GalNAc1* (lane 2), or FLAG-tagged *ST6GalNAc1-short* (lane 3). Detection of bands at just above 55kDa and 69kDa by western blot confirm the successful creation of stable cell lines expressing both isoforms. **C.** Immunofluorescence detection of the sTn antigen using the B72.3 antibody in DU145 cells stably expressing either a control empty vector, full length *ST6GalNAc1* or *ST6GalNAc1-short*. There is increased detection of sTn in cells transfected with both isoforms of *ST6GalNAc1*. Images are representative of 3 independent experimental repeats.

While the 69KDa ST6GalNAc1 protein isoform is known to function as a sialyltransferase [7], no function had been assigned to this shorter protein isoform. To evaluate the relative activity of the 55KDa isoform, FLAG-tagged full-length ST6GalNAc1 and ST6GalNAc1-short were expressed in Flp-In HEK293 cells and purified by immunoprecipitation. Protein purification was confirmed by western blot using antibodies against the FLAG antigen (Figure 4A, upper panel). Empty pcDNA5-FLAG vector was used as a control (lane 1). The activity of the immunoprecipitated proteins were assayed in a sialyltransferase assay. Despite missing the N-terminal 132 amino acids, the shorter isoform of ST6GalNAc1 protein still functioned as a sialyltransferase enzyme at similar levels to the full-length isoform (Figure 4A, lower panel).

To enable us to investigate the relative activities of the short and long ST6GalNAc1 protein isoforms in the absence of competing endogenous protein, we carried out analysis in DU145 cells (RT-qPCR analysis indicated low endogenous levels of *ST6GalNAc1* expression in this cell line) by creating stable cell lines over-expressing each isoform (Figure 4B). Consistent with either ST6GalNAc1 protein isoform being able to transfer a sialic acid in ɑ2-6 linkage onto the Tn antigen, we found that stable cell lines expressing full length ST6GalNAc1 or ST6GalNAc1-short each had increased levels of the sTn antigen (Figure 4C). We also obtained identical results in PC3 cells, where inducible over-expression of both ST6GalNAc1 isoforms increased sTn antigen levels (Supplementary Figure 8).

Taken together, the above data demonstrate that the newly identified short isoform of ST6GalNAc1 is active as a sialyltransferase, and capable of synthesising the cancer-associated sTn antigen similarly to the previously characterised 69KDa ST6GalNAc1 protein isoform. Sub-cellular localisation experiments showed that endogenous ST6GalNAc1 protein (which our western data show will be predominantly the short isoform) is distributed throughout the cytoplasm and specifically in the centrosome in LNCaP cells (Supplementary Figure 9).

### Expression of ST6GalNAc1 in PCa cells reduces adhesion, increases motility and promotes a transition towards a mesenchymal like phenotype

**Figure 5:**
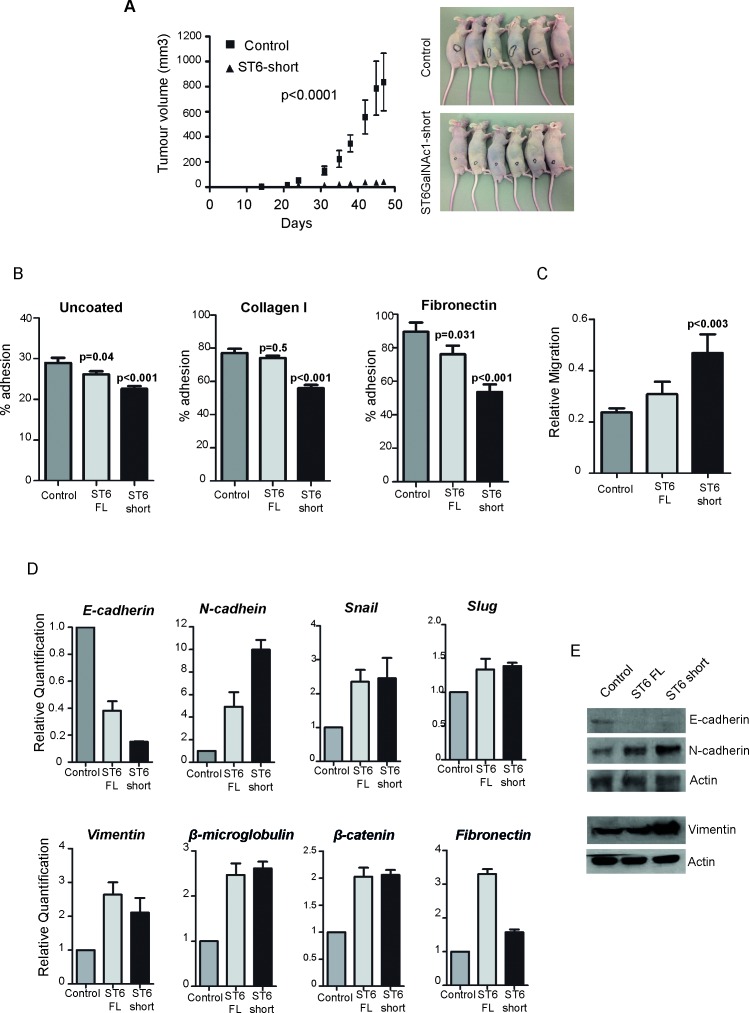
Expression of ST6GalNAc1 in PCa cells reduces adhesion, increases mobility and promotes transition towards a mesenchymal phenotype **A.** Tumour growth curves comparing cells transfected with either empty vector or ST6GalNAc1 (p<0.0001, two-way ANOVA). *Right panel* - mice in both groups - black circles show tumours contours. Adhesion **B.** and migration **C.** assays using our DU145 stable cell lines. There is a significant decrease in adhesion in cells expressing *ST6GalNAc1-short* for uncoated plates and for plates coated with collagen I or fibronectin (*p* **<** 0.001). Cells stably transfected with *ST6GalNAc1-short* also have an increased rate of migration (*p* **<** 0.003). Representative images used for the analysis are shown in Supplementary Figure 8. **D.** Real time PCR analysis of DU145 cells over-expressing *ST6GalNAc1* indicates significant changes in the mRNA expression of key markers of EMT. These included a reduction of *E-cadherin* expression, and an increase in *N-cadherin* expression, as well as an increase in *slug*, *β-microglobulin*, *vimentin* and *β-catenin*. The changes in E-cadherin, N-cadherin and Vimentin expression were confirmed at the protein level by western blot **E.**. Images are representative of 3 independent experimental repeats.

Since expression of ST6GalNAc1 increases tumour mass in breast and colon cancer [20, 21], we analysed the effect of ST6GalNAc1 on prostate tumourigenesis using *in vivo* models. DU145 cells stably transfected with either empty vector control or ST6GalNAc1-short (5 × 10^6^ cells) were injected subcutaneously into nude mice (n=6 mice per group), and the resulting tumours measured twice weekly using a caliper. Tumours grew in 5/6 mice in the control group and 6/6 in the experimental one. Surprisingly, over-expression of ST6GalNAc1-short resulted in a very clear decrease in tumour growth (*p*< 0.0001, two-way ANOVA) (Figure 5A).

Since ST6GalNAc1 expression has been shown to increase establishment of metastases in breast and colon cancer [20, 21], we next examined tumour cell dissemination by injecting DU145 cells tagged with Gaussia luciferase (G-Luc) intravenously (Supplementary Figure 10A). This allows imaging of metastatic foci in vivo but also has the advantage over other luciferase species to be secreted and therefore provide means to monitor disseminated tumour cells by measuring its levels in blood and /or urine [22]. One million cells were injected intravenously (n=6 for each control and experimental group). Whole-body imaging following coelenterazine intraperitoneal administration was performed twice weekly. Over the course of 12-weeks follow-up none of the mice developed clear metastatic foci (representative example in Supplementary Figure 10B). Monitoring of urine and blood in these mice showed G-Luc levels above background (from disseminated cancer cells), however no difference was observed between the control and ST6GalNAc1-short overexpressing cells – for an example see G-Luc quantification in blood at the end-point of the experiment (Supplementary Figure 10C). Our data suggests that unlike in breast and colon cancer, over-expression of ST6GalNAc1 in PCa cells reduces tumour growth and does not affect metastasis. This data would be consistent with a decrease in cell adhesion that would hamper the formation of stable tumour masses.

To investigate how expression of ST6GalNAc1 might result in a decrease in tumour mass, we analysed adhesion and mobility properties of the stably transfected DU145 cells. Over-expression of either isoform of ST6GalNAc1 protein significantly reduced cell adhesion on uncoated plates, and also on plates coated with collagen I and fibronectin (*p*<0.01) (Figure 5B). We also detected an increased rate of cell migration (*p*<0.04) for cells over-expressing both isoforms (Figure 5C and Supplementary Figure 11A). To determine whether this observed phenotypic change was occurring as a result of cellular reprogramming of PCa epithelial cells to a more mesenchymal-like phenotype (a process termed epithelial-mesenchymal transition, or EMT), we examined the expression of key EMT markers in our stable cell lines. RT-qPCR analysis showed that DU145 cells stably over-expressing either isoform of ST6GalNAc1 protein switched from *E-cadherin* to *N-cadherin* gene expression, and also increased expression of *Vimentin, Snail, β-microglobulin* and *β-catenin* (Figure 5D). Loss of E-cadherin and an increase in N-cadherin and vimentin protein expression was confirmed by western blotting (Figure 5E).

The above data indicate stable over-expression of ST6GalNAc1 induces a switch to a more mesenchymal like pattern of gene expression in PCa cells. This predicts that similar changes should also occur in LNCaP cells in response to androgen exposure. Consistent with this, and in agreement with the findings of others [23] we found that culture of LNCaP cells in androgens for 72 hours similarly resulted in a shift towards an more mesenchymal-like pattern of gene expression (Supplementary Table 8). We also observed a significant reduction in cell adhesion in LNCaP cells treated with androgens for 72 hours (*p*=0.015), and this effect was blocked in cells treated with *ST6GalNAc1* esiRNA (Supplementary Figure 11B). Co-expression analysis of *ST6GalNAc1* and EMT markers in the Taylor et al. study [16] also revealed a tendency towards co-occurrence for expression of *ST6GalNAc1* and genes related to EMT (Supplementary Table 9).

### Clinical expression data indicate a changing pattern of *ST6GalNAc1* expression between primary and metastatic tumours

**Figure 6:**
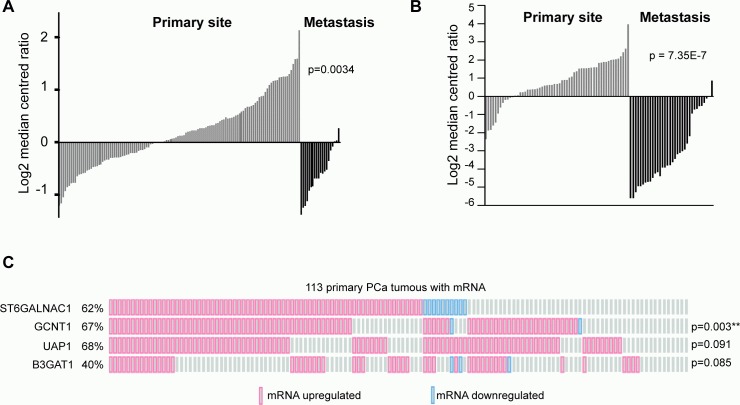
Clinical expression data indicate a changing pattern of *ST6GalNac1* expression between primary and metastatic tumours Expression of *ST6GalNAc1* mRNA in clinical PCa samples measured by **A.** Taylor et al. [16] and **B.** Grasso et al. [10]. In both studies the expression of *ST6GalNAc1* is significantly increased in primary prostate carcinoma, but significantly decreased in metastatic tissue. Data was generated using Oncomine. **C.** Analysis of data generated by Taylor et al. [16] for co-occurrence of *ST6GalNAc1* and other glycosylating enzyme mRNA (Z scores v normal +/− 1.6). *ST6GalNAc1* was significantly co-occurrent with *GCNT1* (*p* = 0.003) (*p* values were derived from exact Fisher tests).

The data presented above suggests that expression of *ST6GalNAc1* mRNA is significantly up-regulated in primary prostate tumours relative to normal prostate gland (Figure 1 and in Supplementary Table 7). Closer inspection of both the Grasso and Taylor datasets revealed a dynamic switch in the expression pattern of *ST6GalNAc1*. A comparison of *ST6GalNAc1* expression in primary and metastatic prostate cancer shows that *ST6GalNAc1* expression strikingly decreases in metastatic PCa cells (Figure 6A, 6B). These results are in agreement with the findings of Varambally [17], where *ST6GalNAc1* was found to be 4.43 fold over-expressed in localised prostate cancer versus benign tissue (*p* = 2.36E-006), but −5.9 fold under-expressed (*p* = 1.85E-007) in metastatic disease versus localised cancer; and are also consistent with a recent study where expression of *ST6GalNAc1* was found to be 2.67 fold reduced in the progression to CRPCa (*p* = 1.55E13) [24].

We also analysed whether expression of *ST6GalNAc1* in clinical data correlates with expression of a variety of glycoprotein synthetic enzymes implicated in PCa progression [25-29]. A recent meta-analysis of clinical PCa gene expression data identified a novel discriminatory signature enriched for 4 glycosylating enzymes *ST6GalNAc1*: glucosaminyl (N-acetyl) transferase 1, core 2 (GCNT1), UDP N-acetylglucosamine pyrophosphorylase 1 (UAP1) and beta-1,3-glucuronyltransferase 1 (B3GAT1) [25]. Analysis of the Taylor et al. dataset [16] revealed that up -regulation of *ST6GalNAc1* is significantly co-occurrent with that of *GCNT1* (*p* = 0.003). We also found a tendency for co-occurrence for *ST6GalNAc1* and *UAP1* (Figure 6C). Hence, 95.7% (67 out of 70) of primary prostate tumours with up-regulated *ST6GalNAc1* mRNA also had up-regulation of at least one other glycosylating enzyme (Z score v normal +/− 1.6). These findings strongly suggest that the *in vivo* impact of gene *ST6GalNAc1* expression in PCa will be influenced by its interplay with other glycosylating enzymes.

## DISCUSSION

Here we show that expression of the sialyltransferase enzyme ST6GalNAc1 is directly activated by androgens in prostate cancer cells. ST6GalNAc1 catalyses synthesis of the cancer-associated sialyl-Tn antigen (sTn), which is expressed in a variety of carcinomas and is correlated with metastasis and poor prognosis in patients [30-33]. Our results show for the first time that expression of sTn is induced by androgens in prostate cancer cells and that this is mediated by ST6GalNAc1. These results are important, as expression of sTn has been detected in up to half of high grade prostate tumours [30, 34].

Surprisingly, we find that rather than the canonical 69 KDa isoform of ST6GalNAc1, a novel ∼55KDa protein isoform of the ST6GalNAc1 protein isoform is instead induced by androgens in prostate cancer cell lines *in vitro*, and also expressed in clinical prostate tissue. This shorter 55 KDa isoform is thus the major expressed ST6GalNAc1 isoform in the prostate. To the best of our knowledge this is the first identification of this shorter ST6GalNAc1protein isoform, which is made from an alternatively spliced mRNA. The canonical 69KDa ST6GalNAc1 protein isoform is the major previously reported isoform in breast and colon cancers where it has been well-studied [7, 21, 31, 35-37]. Despite missing some N-terminal peptide sequence, we find the ∼55KDa protein isoform of the ST6GalNAc1 still functions enzymatically as a sialyltransferase in PCa cells and is capable of synthesising the sTn antigen.

We find that expression of *ST6GalNAc1* in PCa cells changes gene expression towards a more mesenchymal-like pattern, increases cell mobility and decreases cell adhesion. A switch towards a more mesenchymal phenotype has previously been shown to be important for the progress of PCa [38-41], and there is growing evidence for a close link between altered glycosylation and changes in EMT [42-45]. The EMT transition is thought to have a role in the dissemination of tumour cells, however, recent work has suggested that EMT reversion may be required for metastatic outgrowth [46, 47]. Meta-analysis of clinical RNA-Seq datasets show that although *ST6GalNAc1* expression is up-regulated in primary prostate carcinoma, expression is relatively reduced in metastatic prostate tissue. The patterns of *ST6GalNAc1* expression that we report here are thus consistent with a transient role for *ST6GalNAc1* in PCa progression. Our data suggests the increase in *ST6GalNAc1* expression in the primary tumour under the influence of androgens may be the result and not the cause for the initial tumour evolution; however this is a necessary step for tumour cells to be able to evolve to the next level in progression - acquiring a mesenchymal, migratory phenotype. It is interesting to speculate that despite *ST6GalNAc1* being significantly upregulated in primary prostate tissue where it facilitates cell migration from the primary tumour, a reduction in *ST6GalNAc1* may be required to form stable metastases.

Expression of *ST6GalNAc1* in gastric and breast cancer cell lines also leads to reduced cell adhesion and increased cell motility, similar to what we observe in the prostate [20, 21, 31, 48, 49]. However, in breast and colon cancer over-expression of *ST6GalNAc1* has been shown to increase both tumour growth and metastatic ability [20, 21], which was not seen here in PCa cells. Meta-analysis of *ST6GalNAc1* expression in colon and breast cancer tissue versus normal tissue shows significant down-regulation in 6/8 datasets for primary colon cancer (Supplementary Table 7B), and in 4/8 datasets for primary breast cancer (Supplementary Table 7C), the opposite of what we see in PCa. Expression of *ST6GalNAc1* in gastric and breast cancer cell lines induces the sTn antigen and leads to reduced cell adhesion and increased cell motility [20, 21, 31, 48, 49], similar to what we observe in the prostate. Together these data suggest the roles of *ST6GalNAc1* will need to be individually analysed in different cancers.

The androgen-induced changes in sTn expression we detect here most likely occur against a background of androgen-induced changes in cellular glycosylation. There is growing evidence linking changes in the glycoproteome to PCa progression [25-29, 50, 51]. UAP1 and GCNT1 have been shown to be upregulated in PCa [26, 27, 52], and the amino-sugar conjugate, O-linked N-acetylglucosamine (O-GlcNAc) is significantly elevated in prostate cancer tissue [29, 53]. Previous work has shown that the hexosamine biosynthetic pathway enzymes UAP1 and GFPT1 are directly regulated by the AR in PCa cells [27]. Our finding that the O-linked glycosylating enzyme ST6GalNAc1 is androgen-regulated provides further evidence for a direct link between the AR and the glycoproteome in prostate cancer cells, and also suggests that it will be important to understand how these changes function co-ordinately rather than in isolation to modify the cellular behaviour of cancer cells.

## MATERIALS AND METHODS

### RNA-seq and analysis

RNA was prepared for sequencing using a RNA Easy Kit (Qiagen 74104) and treated with DNase 1 (Ambion). RNA-sequencing was carried out using Illumina HiSeq by the Norwegian Sequencing Centre (http://www.sequencing.uio.no/) and analysed as described previously [54]. Reference files for the human genome (UCSC build hg19) were downloaded from the Cufflinks pages: http://cufflinks.cbcb.umd.edu/igenomes.html". UCSC-hg19 package from June 2012 was used. The software versions used for the analysis were TopHat v1.4.1, SAM tools v0.1.18 (r982:295), bowtie v0.12.8 (64-bit) and cufflinks v1.3.0 (linked against Boost v104000). Nature protocol published under doi:10.1038/nprot.2012.016 was followed. For steps 1-5, no parameters (except for paths to input/output files) were altered. In step 15, additional switches -s, -R, and -C were used when running cuffcompare. Steps 16-18 (extraction of significant results) were performed on the command line. The complete command list for the individual steps is given in Supplementary Methods 1.

### Antibodies

The following antibodies were used: anti-ST6GalNAc1 C-term (Rb pAb, 15363-1, Proteintech), anti- ST6GalNAc1 N-term (Rb pAb, HPA014975, Sigma-Aldrich), anti-Sialyl Tn (Ms mAb, Abcam 115957), anti-CDK5RAP2 (Rb pAb, ab86340, Abcam), γ-tubulin [GTU-88] (Ms mAb, ab11316, Abcam), α-tubulin (Ms mAB, T6199, Sigma), Lectin PNA [Alexa Fluor^®^ 488 conjugated] (L21409, Life Technologies), anti-actin (Rb pAb, A2668, Sigma), anti-FLAG mouse monoclonal antibody (F3165, Sigma), normal rabbit IgG (711-035-152 Jackson labs) and normal mouse IgG (715-036-150 Jackson labs). Anti-sialyl Tn B72.3 (mAb) was prepared as follows: The B72.3 hybridoma cell line [55] was obtained from ATCC. The hybridoma cells were adapted to serum-free medium (Hybridoma SFM, Invitrogen). B72.3 mAb was affinity-purified from the tissue culture supernatant using the AbSelect mouse TCS purification system (Innova Biosciences).

### DNA constructs

Full length ST6GalNAc1 cloned into pcDNA3.1+ (V790-20, Invitrogen) was a kind gift from Hidenori Ozaki (AIST, Japan). This was then cloned into pX3FLAG CMV 10 (E7658, Sigma) using EcoRI and XbaI and into pcDNA5-FLAG (V6520-20, Invitrogen) using BamH1 and XhoI. ST6GalNAc1 short isoform was cloned into pX3FLAG CMV 10 (E7658, Sigma) using EcoR1 and XbaI and into pcDNA5-FLAG (V6520-20, Invitrogen) using BamH1 and XhoI. Full length ST6GalNAc1-GFP and ST6GalNAc1 short-GFP were cloned into pcDNA3.1+ using EcoR1 and XhoI.

### Cell culture

Cell culture was as described previously [11, 56, 57]. All cells were grown at 37°C in 5% CO_2_. LNCaP cells (CRL-1740, ATCC) were maintained in RPMI-1640 with L-Glutamine (PAA Laboratories, R15-802) supplemented with 10% Fetal Bovine Serum (FBS) (PAA Laboratories, A15-101). For androgen treatment of cells, medium was supplemented with 10% dextran charcoal stripped FBS (PAA Laboratories, A15-119) to produce a steroid-deplete medium. Following culture for 72 hours, 10 nM synthetic androgen analogue methyltrienolone (R1881) (Perkin-Elmer, NLP005005MG) was added (Androgen +) or absent (Steroid deplete) for the times indicated. Where indicated, LNCaP cells were pre-treated with 10 μm bicalutamide (Casodex, AstraZeneca) or ethanol (vehicle) for 2 hours prior to addition of 10 nM R1881 for the times indicated. VCaP (ATCC^®^ CRL-2876), PC-3 (CRL-1435, ATCC), PC-3M [58], CWR22Rv1 (CRL-2505, ATCC), DU145 (HTB-81, ATCC), and BPH-1 cells [59] were maintained in RPMI-1640 with L-Glutamine supplemented with 10% FBS. LNCaP-AI and LNCaP-cdxR were derived from LNCaP parental cells and maintained as previously described [60, 61]. Transient transfections of GFP-tagged proteins were carried out using Lipofectamine 2000 (11668-027, Invitrogen). Stable DU145 cell lines expressing full length *ST6GalNAc1 and ST6GalNAc1-short* and were generated using DNA constructs cloned into pX3FLAG CMV 10 (E7658, Sigma). Cells were transfected using Lipofectamine 2000 followed by selection with 300μg/ml Geneticin (Invitrogen, 10131019) (reduced to 150μg/ml following the death of untransfected cells) for at least four weeks. Flp-In™-293 cells (R750-07, Invitrogen) were maintained in DMEM GlutaMax (Invitrogen, 10566-040), supplemented with 10% FBS (PAA Laboratories, A15-101) and stable cell lines generated using the Flp-In T-Rex Core Kit (K6500-01, Invitrogen) according to the manufacturer's instructions (DNA constructs were cloned into pcDNA5-FLAG (V6520-20, Invitrogen)). Protein expression was induced using 1 μg/ml tetracycline (T7660, Sigma) for 72 hours. PC3 inducible cell lines were created using the T-REx™ System according to the manufacturer's instructions (Invitogen, K102001), and induced with tetracycline as above.

### ChIP

Chromatin of LNCaP and VCaP cells was immunoprecipitated as previously described [62] using AR antibody (N-20X, Santa Cruz). Levels of ChIP enrichment were determined by SYBR green quantitative PCR. Six previously reported [3] AR binding sites both upstream and at *ST6GalNAc1* were assessed for AR enrichments. All primer sequences are listed in Supplementary Table 1.

### Clinical Samples

Our study made use of RNA from 32 benign samples from patients with benign prostatic hyperplasia (BPH) and 17 malignant samples from transurethral resection of the prostate (TURP) samples. Malignant status and Gleason score were obtained for these patients by histological analysis. We also analysed normal and matched PCa tissue from 9 patients obtained by radical prostatectomy. The samples were obtained with ethical approval through the Exeter NIHR Clinical Research Facility tissue bank (Ref: STB20). We also examined RNA and protein lysates from five clinical PCa tissues. For the use of these samples, full ethical approval was obtained from the Northumberland, Tyne and Wear NHS Strategic Health Authority Local Research Ethics Committee (Ref: 2003/11). Written informed consent for the use of surgically obtained tissue was provided by all patients.

### RT-qPCR

Cells were harvested and total RNA extracted using TRIzol (Invitrogen, 15596-026) according to manufacturer's instructions. RNA was treated with DNase 1 (Ambion) and cDNA was generated by reverse transcription of 1μg of total RNA using the Superscript VILO cDNA synthesis kit (Invitrogen, 11754-050). Quantitative PCR (qPCR) (Applied Biosystems 7900HT) was performed in triplicate on cDNA using SYBR^®^ Green PCR Master Mix (Invitrogen, 4309155). Samples were normalised using the average of three reference genes, GAPDH, β -tubulin and actin. All primer sequences are listed in Supplementary Table 1.

### siRNA

Knockdown of *ST6GalNAc1* was carried out using a pre-designed silencer select siRNA (Ambion 4392420) and further confirmed using *ST6GalNAc1* esiRNA from Sigma-Aldrich (EHU133871). Scrambled silencer select siRNA was used as a control (Ambion). AR esiRNA was obtained from Sigma-Aldrich (EHU025951).

### Sialyltransferase assay

FLAG-tagged ST6GalNAc1 full length and ST6GalNAc1-short were immunoprecipitated from Flp-In™-293 cell lysates using EZview Red ANTI-FLAG M2 Affinity gel (Sigma F2426). Sialyltransferase assays were performed using the Sialyltransferase Activity Kit (R&D Systems EA002) according to the manufacturer's instructions using 25nmol of CMP-Neu5Ac (Sigma), 0.5mg asialofetuin (Sigma) and 50ng of Coupling Phosphatise 2 for 20 minutes at 37°C. Phosphate Standard (Part 895408) was used as a positive control and to produce a standard curve.

### Adhesion assays

Coated plates were purchased from R&D systems. Cells were labelled with 5μM Calcein-AM (BD Biosciences). 50,000 cells per well were allowed to adhere for two hours, washed 4 times with PBS, and the absorbance measured at 485/538nm. The percentage adhesion was then calculated by comparing with an identical unwashed plate.

### Migration assay

Migration assays were carried out using an Oris^TM^ 96 well Cell Migration Assay (Platypus Technologies CMA1.101) using 100,000 cells per well. Cells were labelled with Calein AM and allowed to adhere for 4 hours, before removal of the seeding stoppers. Migration of cells into the centre of each well after 15 hours was measured using ImageJ.

### *In vivo* tumour growth and metastasis assays

DU145 cell stably transfected with either empty vector control or ST6GalNAc1-short were injected subcutaneously in nude mice (5 × 10^6^ for each mouse, *n***=** 6 mice in each group). Once tumours became visible their width and length was measured twice weekly using a caliper. When the first tumour reached the maximum allowed size under the UK Home Office project licence (12 mm in any diameter) all mice were culled. Tumour volumes were calculated using the formula: [length × width × (length+width)/2].

For metastasis experiments, cells were transduced with a lentivirus expressing Gaussia luciferase (GeneCopoeia LPP-MGLUC-LV105-025) and selected using puromycin (1 ug/ml). Luciferase was detected using the BioLux^®^ Gaussia Luciferase Assay Kit (NEB, E3300S) according to the manufacturer's instructions, and normalised to the relative cell number for each cell line. One million cells were administered intravenously by retroorbital injection. To assess metastatic spread, mice were imaged twice weekly using a Xenogen *in vivo* imaging device following intraperitoneal injections with 200 microliters coelenterazine (RediJect, Perkin Elmer). For measuring levels of Gaussia luciferase in urine or blood, 10 microliters of urine was collected directly from urethral openings. Blood was collected by pricking the tail-vein and immediately dispensing into an EDTA-coated tube. Both fluids were pipetted into a 96-well plate, mixed with 100 microliters coelenterazine and imaged in the Xenogen devide sequentially at 5, 10, 15 minutes; the peak signal (at 10 minutes) was quantified.

## SUPPLEMENTARY MATERIAL FIGURES AND TABLES





















## References

[1] Sharma NL, Massie CE, Ramos-Montoya A, Zecchini V, Scott HE, Lamb AD, MacArthur S, Stark R, Warren AY, Mills IG, Neal DE (2013). The androgen receptor induces a distinct transcriptional program in castration-resistant prostate cancer in man. Cancer Cell.

[2] Mills IG (2014). Maintaining and reprogramming genomic androgen receptor activity in prostate cancer. Nat Rev Cancer.

[3] Massie CE, Lynch A, Ramos-Montoya A, Boren J, Stark R, Fazli L, Warren A, Scott H, Madhu B, Sharma N, Bon H, Zecchini V, Smith D-M, DeNicola GM, Mathews N, Osborne M (2011). The androgen receptor fuels prostate cancer by regulating central metabolism and biosynthesis. EMBO J.

[4] Takano R, Muchmore E, Dennis JW (1994). Sialylation and malignant potential in tumour cell glycosylation mutants. Glycobiology.

[5] Hakomori S (1985). Aberrant glycosylation in cancer cell membranes as focused on glycolipids: overview and perspectives. Cancer Res.

[6] Hakomori S (2002). Glycosylation defining cancer malignancy: new wine in an old bottle. Proc Natl Acad Sci U S A.

[7] Ikehara Y, Kojima N, Kurosawa N, Kudo T, Kono M, Nishihara S, Issiki S, Morozumi K, Itzkowitz S, Tsuda T, Nishimura SI, Tsuji S, Narimatsu H (1999). Cloning and expression of a human gene encoding an N-acetylgalactosamine-alpha2,6-sialyltransferase (ST6GalNAc I): a candidate for synthesis of cancer-associated sialyl-Tn antigens. Glycobiology.

[8] Khabaz MN, McClure J, McClure S, Stoddart RW (2010). Glycophenotype of prostatic carcinomas. Folia Histochem Cytobiol.

[9] Horoszewicz JS, Leong SS, Kawinski E, Karr JP, Rosenthal H, Chu TM, Mirand EA, Murphy GP (1983). LNCaP model of human prostatic carcinoma. Cancer Res.

[10] Grasso CS, Wu YM, Robinson DR, Cao X, Dhanasekaran SM, Khan AP, Quist MJ, Jing X, Lonigro RJ, Brenner JC, Asangani IA, Ateeq B, Chun SY, Siddiqui J, Sam L, Anstett M (2012). The mutational landscape of lethal castration-resistant prostate cancer. Nature.

[11] Rajan P, Dalgliesh C, Carling PJ, Buist T, Zhang C, Grellscheid SN, Armstrong K, Stockley J, Simillion C, Gaughan L, Kalna G, Zhang MQ, Robson CN, Leung HY, Elliott DJ (2011). Identification of novel androgen-regulated pathways and mRNA isoforms through genome-wide exon-specific profiling of the LNCaP transcriptome. PLoS One.

[12] Ho LL, Kench JG, Handelsman DJ, Scheffer GL, Stricker PD, Grygiel JG, Sutherland RL, Henshall SM, Allen JD, Horvath LG (2008). Androgen regulation of multidrug resistance-associated protein 4 (MRP4/ABCC4) in prostate cancer. Prostate.

[13] Lapointe J, Li C, Higgins JP, van de Rijn M, Bair E, Montgomery K, Ferrari M, Egevad L, Rayford W, Bergerheim U, Ekman P, DeMarzo AM, Tibshirani R, Botstein D, Brown PO, Brooks JD (2004). Gene expression profiling identifies clinically relevant subtypes of prostate cancer. Proc Natl Acad Sci U S A.

[14] Vanaja DK, Cheville JC, Iturria SJ, Young CY (2003). Transcriptional silencing of zinc finger protein 185 identified by expression profiling is associated with prostate cancer progression. Cancer Res.

[15] Arredouani MS, Lu B, Bhasin M, Eljanne M, Yue W, Mosquera JM, Bubley GJ, Li V, Rubin MA, Libermann TA, Sanda MG (2009). Identification of the transcription factor single-minded homologue 2 as a potential biomarker and immunotherapy target in prostate cancer. Clin Cancer Res.

[16] Taylor BS, Schultz N, Hieronymus H, Gopalan A, Xiao Y, Carver BS, Arora VK, Kaushik P, Cerami E, Reva B, Antipin Y, Mitsiades N, Landers T, Dolgalev I, Major JE, Wilson M (2010). Integrative genomic profiling of human prostate cancer. Cancer Cell.

[17] Varambally S, Yu J, Laxman B, Rhodes DR, Mehra R, Tomlins SA, Shah RB, Chandran U, Monzon FA, Becich MJ, Wei JT, Pienta KJ, Ghosh D, Rubin MA, Chinnaiyan AM (2005). Integrative genomic and proteomic analysis of prostate cancer reveals signatures of metastatic progression. Cancer Cell.

[18] Makkonen H, Kauhanen M, Jaaskelainen T, Palvimo JJ (2011). Androgen receptor amplification is reflected in the transcriptional responses of Vertebral-Cancer of the Prostate cells. Mol Cell Endocrinol.

[19] Uhlen M, Fagerberg L, Hallstrom BM, Lindskog C, Oksvold P, Mardinoglu A, Sivertsson A, Kampf C, Sjostedt E, Asplund A, Olsson I, Edlund K, Lundberg E, Navani S, Szigyarto CA, Odeberg J (2015). Proteomics. Tissue-based map of the human proteome. Science.

[20] Julien S, Adriaenssens E, Ottenberg K, Furlan A, Courtand G, Vercoutter-Edouart AS, Hanisch FG, Delannoy P, Le Bourhis X (2006). ST6GalNAc I expression in MDA-MB-231 breast cancer cells greatly modifies their O-glycosylation pattern and enhances their tumourigenicity. Glycobiology.

[21] Ozaki H, Matsuzaki H, Ando H, Kaji H, Nakanishi H, Ikehara Y, Narimatsu H (2012). Enhancement of metastatic ability by ectopic expression of ST6GalNAcI on a gastric cancer cell line in a mouse model. Clin Exp Metastasis.

[22] Chung E, Yamashita H, Au P, Tannous BA, Fukumura D, Jain RK (2009). Secreted Gaussia luciferase as a biomarker for monitoring tumor progression and treatment response of systemic metastases. PLoS One.

[23] Zhu ML, Kyprianou N (2010). Role of androgens and the androgen receptor in epithelial-mesenchymal transition and invasion of prostate cancer cells. FASEB journal : official publication of the Federation of American Societies for Experimental Biology.

[24] Tamura K, Furihata M, Tsunoda T, Ashida S, Takata R, Obara W, Yoshioka H, Daigo Y, Nasu Y, Kumon H, Konaka H, Namiki M, Tozawa K, Kohri K, Tanji N, Yokoyama M (2007). Molecular features of hormone-refractory prostate cancer cells by genome-wide gene expression profiles. Cancer Res.

[25] Barfeld SJ, East P, Zuber V, Mills IG (2014). Meta-analysis of prostate cancer gene expression data identifies a novel discriminatory signature enriched for glycosylating enzymes. BMC Med Genomics.

[26] Chen Z, Gulzar ZG, St Hill CA, Walcheck B, Brooks JD (2014). Increased expression of GCNT1 is associated with altered O-glycosylation of PSA, PAP, and MUC1 in human prostate cancers. Prostate.

[27] Itkonen HM, Engedal N, Babaie E, Luhr M, Guldvik IJ, Minner S, Hohloch J, Tsourlakis MC, Schlomm T, Mills IG (2015). UAP1 is overexpressed in prostate cancer and is protective against inhibitors of N-linked glycosylation. Oncogene.

[28] Itkonen HM, Mills IG (2013). N-linked glycosylation supports cross-talk between receptor tyrosine kinases and androgen receptor. PLoS One.

[29] Kamigaito T, Okaneya T, Kawakubo M, Shimojo H, Nishizawa O, Nakayama J (2014). Overexpression of O-GlcNAc by prostate cancer cells is significantly associated with poor prognosis of patients. Prostate cancer and prostatic diseases.

[30] Genega EM, Hutchinson B, Reuter VE, Gaudin PB (2000). Immunophenotype of high-grade prostatic adenocarcinoma and urothelial carcinoma. Mod Pathol.

[31] Julien S, Krzewinski-Recchi MA, Harduin-Lepers A, Gouyer V, Huet G, Le Bourhis X, Delannoy P (2001). Expression of sialyl-Tn antigen in breast cancer cells transfected with the human CMP-Neu5Ac: GalNAc alpha2,6-sialyltransferase (ST6GalNac I) cDNA. Glycoconjugate journal.

[32] Kobayashi H, Terao T, Kawashima Y (1992). Serum sialyl Tn antigen as a prognostic marker in patients with epithelial ovarian cancer. Nihon Sanka Fujinka Gakkai Zasshi.

[33] Pinho S, Marcos NT, Ferreira B, Carvalho AS, Oliveira MJ, Santos-Silva F, Harduin-Lepers A, Reis CA (2007). Biological significance of cancer-associated sialyl-Tn antigen: modulation of malignant phenotype in gastric carcinoma cells. Cancer Lett.

[34] Myers RB, Meredith RF, Schlom J, LoBuglio AF, Bueschen AJ, Wheeler RH, Stockard CR, Grizzle WE (1994). Tumor associated glycoprotein-72 is highly expressed in prostatic adenocarcinomas. J Urol.

[35] Marcos NT, Bennett EP, Gomes J, Magalhaes A, Gomes C, David L, Dar I, Jeanneau C, DeFrees S, Krustrup D, Vogel LK, Kure EH, Burchell J, Taylor-Papadimitriou J, Clausen H, Mandel U (2011). ST6GalNAc-I controls expression of sialyl-Tn antigen in gastrointestinal tissues. Frontiers in bioscience.

[36] Sewell R, Backstrom M, Dalziel M, Gschmeissner S, Karlsson H, Noll T, Gatgens J, Clausen H, Hansson GC, Burchell J, Taylor-Papadimitriou J (2006). The ST6GalNAc-I sialyltransferase localizes throughout the Golgi and is responsible for the synthesis of the tumor-associated sialyl-Tn O-glycan in human breast cancer. J Biol Chem.

[37] Tamura F, Sato Y, Hirakawa M, Yoshida M, Ono M, Osuga T, Okagawa Y, Uemura N, Arihara Y, Murase K, Kawano Y, Iyama S, Takada K, Hayashi T, Sato T, Miyanishi K (2014). RNAi-mediated gene silencing of ST6GalNAc I suppresses the metastatic potential in gastric cancer cells. Gastric Cancer.

[38] Gravdal K, Halvorsen OJ, Haukaas SA, Akslen LA (2007). A switch from E-cadherin to N-cadherin expression indicates epithelial to mesenchymal transition and is of strong and independent importance for the progress of prostate cancer. Clin Cancer Res.

[39] Tran NL, Nagle RB, Cress AE, Heimark RL (1999). N-Cadherin expression in human prostate carcinoma cell lines. An epithelial-mesenchymal transformation mediating adhesion withStromal cells. Am J Pathol.

[40] Umbas R, Isaacs WB, Bringuier PP, Schaafsma HE, Karthaus HF, Oosterhof GO, Debruyne FM, Schalken JA (1994). Decreased E-cadherin expression is associated with poor prognosis in patients with prostate cancer. Cancer Res.

[41] Wei J, Xu G, Wu M, Zhang Y, Li Q, Liu P, Zhu T, Song A, Zhao L, Han Z, Chen G, Wang S, Meng L, Zhou J, Lu Y, Ma D (2008). Overexpression of vimentin contributes to prostate cancer invasion and metastasis via src regulation. Anticancer Res.

[42] Lu J, Isaji T, Im S, Fukuda T, Hashii N, Takakura D, Kawasaki N, Gu J (2014). beta-Galactoside alpha2,6-sialyltranferase 1 promotes transforming growth factor-beta-mediated epithelial-mesenchymal transition. J Biol Chem.

[43] Freire-de-Lima L (2014). Sweet and sour: the impact of differential glycosylation in cancer cells undergoing epithelial-mesenchymal transition. Front Oncol.

[44] Freire-de-Lima L, Gelfenbeyn K, Ding Y, Mandel U, Clausen H, Handa K, Hakomori SI (2011). Involvement of O-glycosylation defining oncofetal fibronectin in epithelial-mesenchymal transition process. Proc Natl Acad Sci U S A.

[45] Ding Y, Gelfenbeyn K, Freire-de-Lima L, Handa K, Hakomori SI (2012). Induction of epithelial-mesenchymal transition with O-glycosylated oncofetal fibronectin. FEBS Lett.

[46] Ocana OH, Corcoles R, Fabra A, Moreno-Bueno G, Acloque H, Vega S, Barrallo-Gimeno A, Cano A, Nieto MA (2012). Metastatic colonization requires the repression of the epithelial-mesenchymal transition inducer Prrx1. Cancer Cell.

[47] Tsai JH, Donaher JL, Murphy DA, Chau S, Yang J (2012). Spatiotemporal regulation of epithelial-mesenchymal transition is essential for squamous cell carcinoma metastasis. Cancer Cell.

[48] Julien S, Lagadec C, Krzewinski-Recchi MA, Courtand G, Le Bourhis X, Delannoy P (2005). Stable expression of sialyl-Tn antigen in T47-D cells induces a decrease of cell adhesion and an increase of cell migration. Breast Cancer Res Treat.

[49] Marcos NT, Pinho S, Grandela C, Cruz A, Samyn-Petit B, Harduin-Lepers A, Almeida R, Silva F, Morais V, Costa J, Kihlberg J, Clausen H, Reis CA (2004). Role of the human ST6GalNAc-I and ST6GalNAc-II in the synthesis of the cancer-associated sialyl-Tn antigen. Cancer Res.

[50] Ma W, Diep K, Fritsche HA, Shore N, Albitar M (2014). Diagnostic and prognostic scoring system for prostate cancer using urine and plasma biomarkers. Genetic testing and molecular biomarkers.

[51] Lynch TP, Ferrer CM, Jackson SR, Shahriari KS, Vosseller K, Reginato MJ (2012). Critical role of O-Linked beta-N-acetylglucosamine transferase in prostate cancer invasion, angiogenesis, and metastasis. J Biol Chem.

[52] Hagisawa S, Ohyama C, Takahashi T, Endoh M, Moriya T, Nakayama J, Arai Y, Fukuda M (2005). Expression of core 2 beta1,6-N-acetylglucosaminyltransferase facilitates prostate cancer progression. Glycobiology.

[53] Itkonen HM, Minner S, Guldvik IJ, Sandmann MJ, Tsourlakis MC, Berge V, Svindland A, Schlomm T, Mills IG (2013). O-GlcNAc transferase integrates metabolic pathways to regulate the stability of c-MYC in human prostate cancer cells. Cancer Res.

[54] Trapnell C, Roberts A, Goff L, Pertea G, Kim D, Kelley DR, Pimentel H, Salzberg SL, Rinn JL, Pachter L (2012). Differential gene and transcript expression analysis of RNA-seq experiments with TopHat and Cufflinks. Nat Protoc.

[55] Kjeldsen T, Clausen H, Hirohashi S, Ogawa T, Iijima H, Hakomori S (1988). Preparation and characterization of monoclonal antibodies directed to the tumor-associated O-linked sialosyl-2——6 alpha-N-acetylgalactosaminyl (sialosyl-Tn) epitope. Cancer Res.

[56] Munkley J, Rajan P, Lafferty NP, Dalgliesh C, Jackson RM, Robson CN, Leung HY, Elliott DJ (2014). A novel androgen-regulated isoform of the TSC2 tumour suppressor gene increases cell proliferation. Oncotarget.

[57] Munkley J, Lafferty NP, Kalna G, Robson CN, Leung HY, Rajan P, Elliott DJ (2015). Androgen-regulation of the protein tyrosine phosphatase PTPRR activates ERK1/2 signalling in prostate cancer cells. BMC Cancer.

[58] Kozlowski JM, Fidler IJ, Campbell D, Xu ZL, Kaighn ME, Hart IR (1984). Metastatic behavior of human tumor cell lines grown in the nude mouse. Cancer Res.

[59] Hayward SW, Dahiya R, Cunha GR, Bartek J, Deshpande N, Narayan P (1995). Establishment and characterization of an immortalized but non-transformed human prostate epithelial cell line: BPH-1. *In Vitro* Cell Dev Biol Anim.

[60] Halkidou K, Gnanapragasam VJ, Mehta PB, Logan IR, Brady ME, Cook S, Leung HY, Neal DE, Robson CN (2003). Expression of Tip60, an androgen receptor coactivator, and its role in prostate cancer development. Oncogene.

[61] Rigas AC, Robson CN, Curtin NJ (2007). Therapeutic potential of CDK inhibitor NU2058 in androgen-independent prostate cancer. Oncogene.

[62] Forsberg EC, Downs KM, Christensen HM, Im H, Nuzzi PA, Bresnick EH (2000). Developmentally dynamic histone acetylation pattern of a tissue-specific chromatin domain. Proc Natl Acad Sci U S A.

[63] Rajan P, Sudbery IM, Villasevil ME, Mui E, Fleming J, Davis M, Ahmad I, Edwards J, Sansom OJ, Sims D, Ponting CP, Heger A, McMenemin RM, Pedley ID, Leung HY (2014). Next-generation sequencing of advanced prostate cancer treated with androgen-deprivation therapy. Eur Urol.

