# Supplementary material for: The androgen receptor controls expression of the cancer-associated sTn antigen and cell adhesion through induction of ST6GalNAc1 in prostate cancer
Source: Oncotarget. 2015 Oct 7;6(33):34358–74. doi: 10.18632/oncotarget.6024 (PMC4741458; doi:10.18632/oncotarget.6024)
Supplement: Supplementary file 1 [file oncotarget-06-34358-s001.pdf]

The androgen receptor controls expression of the cancer-associated sTn antigen and cell adhesion through induction of ST6GalNAc1 in prostate cancer

Supplementary Material

(A) Expression level distibution plot

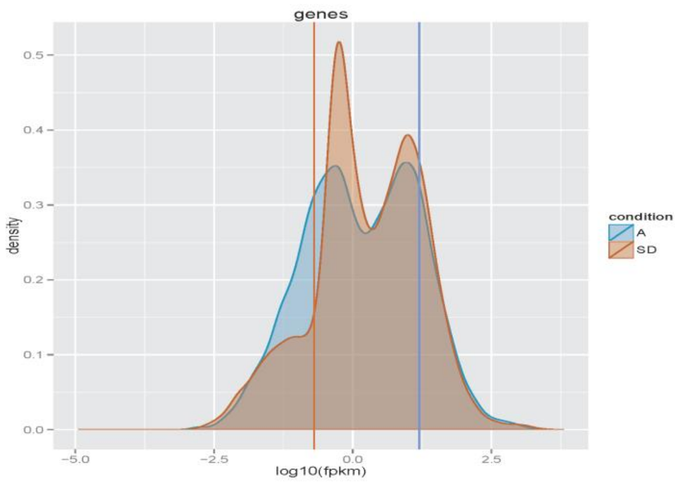

(B) Expression Scatter plot

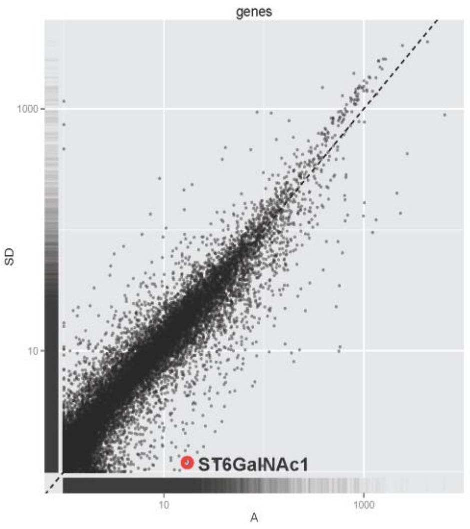

(C) Expression Volcano plot

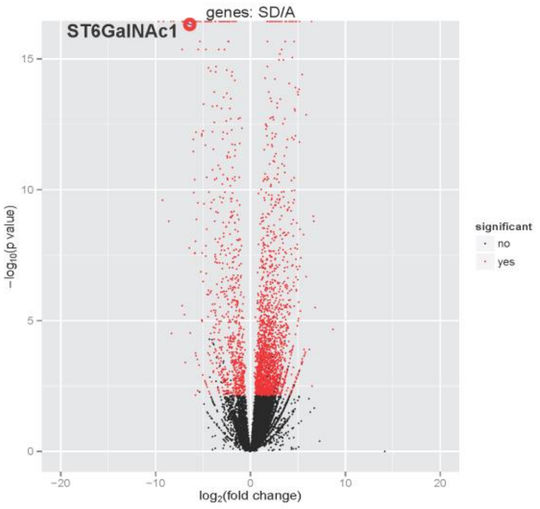

Supplementary Figure 1: Volcano plot and expression distribution plots of RNA sequencing data.

(A) Expression level distributions for all genes, plotted separately for steroid deplete cells (brown) and androgen treated cells (blue). Expression levels for the *ST6GALNAC1* gene are marked with vertical lines of corresponding colours (x-coordinates -0.6960807 and 1.198445. The x-coordinate values are log10 values of the fpkm expression values for ST6GALNAC1: log10(15.792300) = 1.198445 (A+), log10(0.201335) = -0.6960807 (SD). (B) Scatter plot of expression values (fpkm values with addition of 1) for individual genes in the two tested conditions. Genes with similar expression levels in the two conditions lie close to the dashed diagonal line. Added are a smooth-fit regression line (full, blueline) and a red highlighting circle for the ST6GALNAC1 gene (coordinates [16.7923, 1.201335])." Notes: the coordinates are computed as 15.792300 + 1 = 16.7923 (condition A), 0.201335 + 1 = 1.201335 (condition SD). (C) Volcano plot displaying (in red) genes with significant (q_value <= 0.05) differential expression between the two tested conditions. The ST6GALNAC1 gene is highlighted with a red circle (coordinates [-6.29347, "infinity"])." Notes: the x-coordinate is the log2_fold_change in expression between conditions A+ and SD: log2(0.201335/15.7923) = -6.29347; the y-coordinate is the −log10 value of the p-value for significant differential gene expression: −log10(0) = infinity.

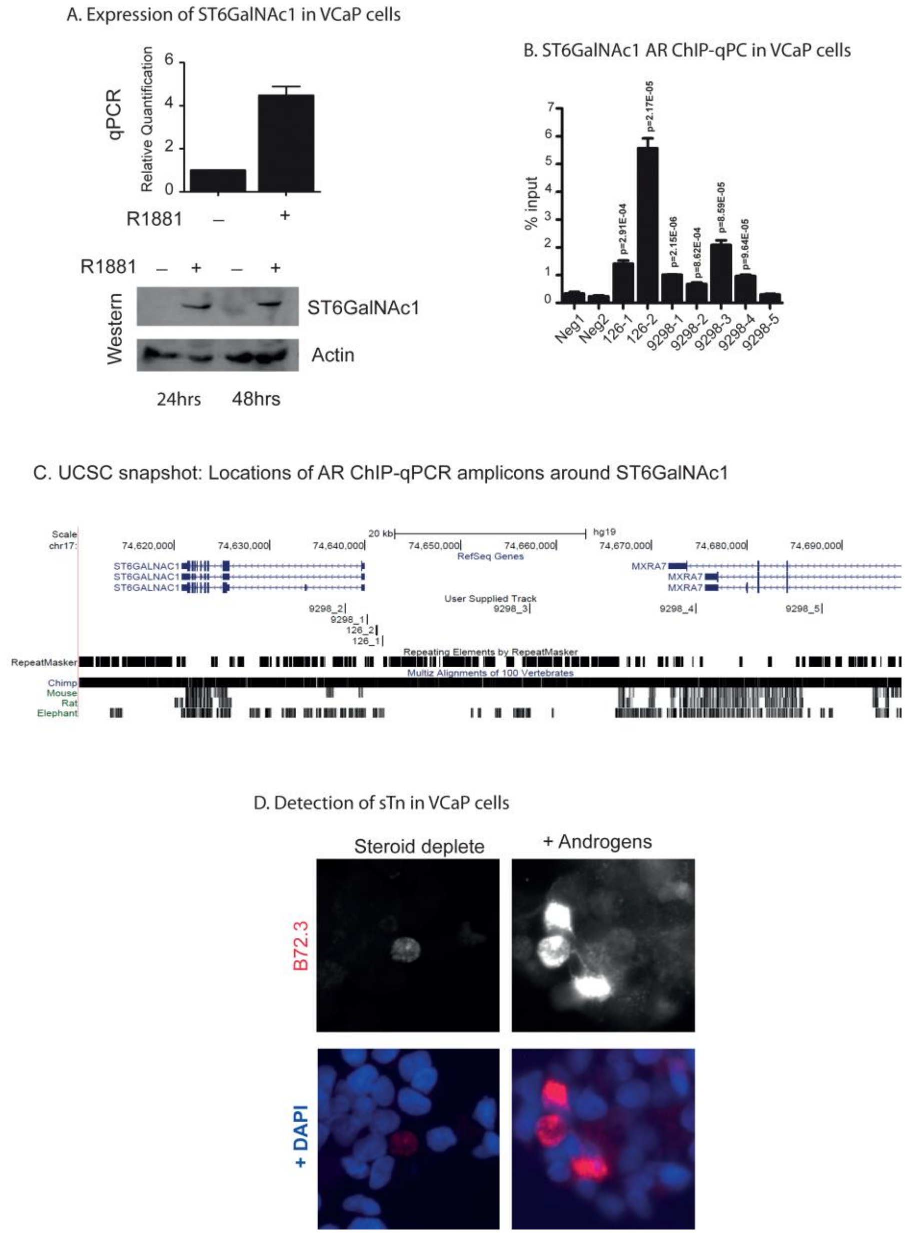

Supplementary Figure 2

(A) Expression of ST6GalNAc1 in VCaP cells. Treatment of cells with 10nM R1881 for 24 hours induces expression of ST6GalNAc1 mRNA (detected by qPCR). By 48 hours we also detect a 55kDa band corresponding to ST6GalNAc1 by western blot. (B) AR-ChIP in VCaP cells revealed 6 AR binding sites in close proximity to the *ST6GalNAc1* gene. (C) UCSC snapshot: Locations of AR binding sites around *ST6GalNAc1.* (D) Detection of sTn antigen using the B72.3 antibody in VCaP cells grown in the absence (steroid deplete) or presence of 10nM R1881 (+androgens) for 72 hours. Bar is 10µm.

ST6GalNAc2-6 mRNA expression in LNCaP cells grown with (A+) and without (SD) 10nM R1881 for 24hrs

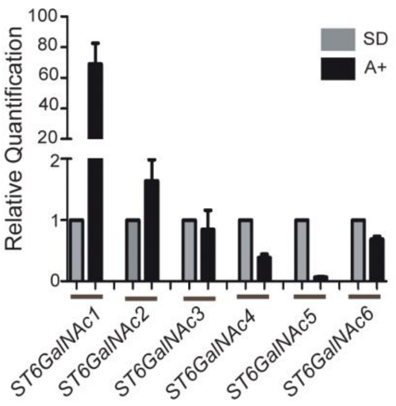

Androgen induction of *ST6GalNac1* was very specific: no parallel induction of the *ST6GalNAc2-6* genes was observed in response to androgen exposure. However, we did observe a reduction in expression of *ST6GalNac4 and 5*. ST6GalNAc4 has been linked to production of the Thomsen-Friedenreich antigen [1] and ST6GalNAc5 has been shown to synthesise alpha-series gangliosides [2]. However, induction of the sTn antigen has been shown to be specific to ST6GalNAc1 [3].

1. Reticker-Flynn, N.E. and S.N. Bhatia, *Aberrant glycosylation promotes lung cancer metastasis through adhesion to galectins in the metastatic niche.* Cancer Discov, 2015. **5**(2): p. 168-81.
2. Tsuchida, A., et al., *Synthesis of disialyl Lewis a (Le(a)) structure in colon cancer cell lines by a sialyltransferase, ST6GalNAc VI, responsible for the synthesis of alpha-series gangliosides.* J Biol Chem, 2003. **278**(25): p. 22787-94.
3. Marcos, N.T., et al., *ST6GalNAc-I controls expression of sialyl-Tn antigen in gastrointestinal tissues.* Front Biosci (Elite Ed), 2011. **3**: p. 1443-55.

Supplementary Figure 3

Real-time PCR analysis of expression of *ST6GalNAc1-6* indicated that no other members of the family are significantly up-regulated by treatment with 10 nM R1881 for 24 hours. These results underscore the early and direct regulation of *ST6GalNAc1* by the AR.

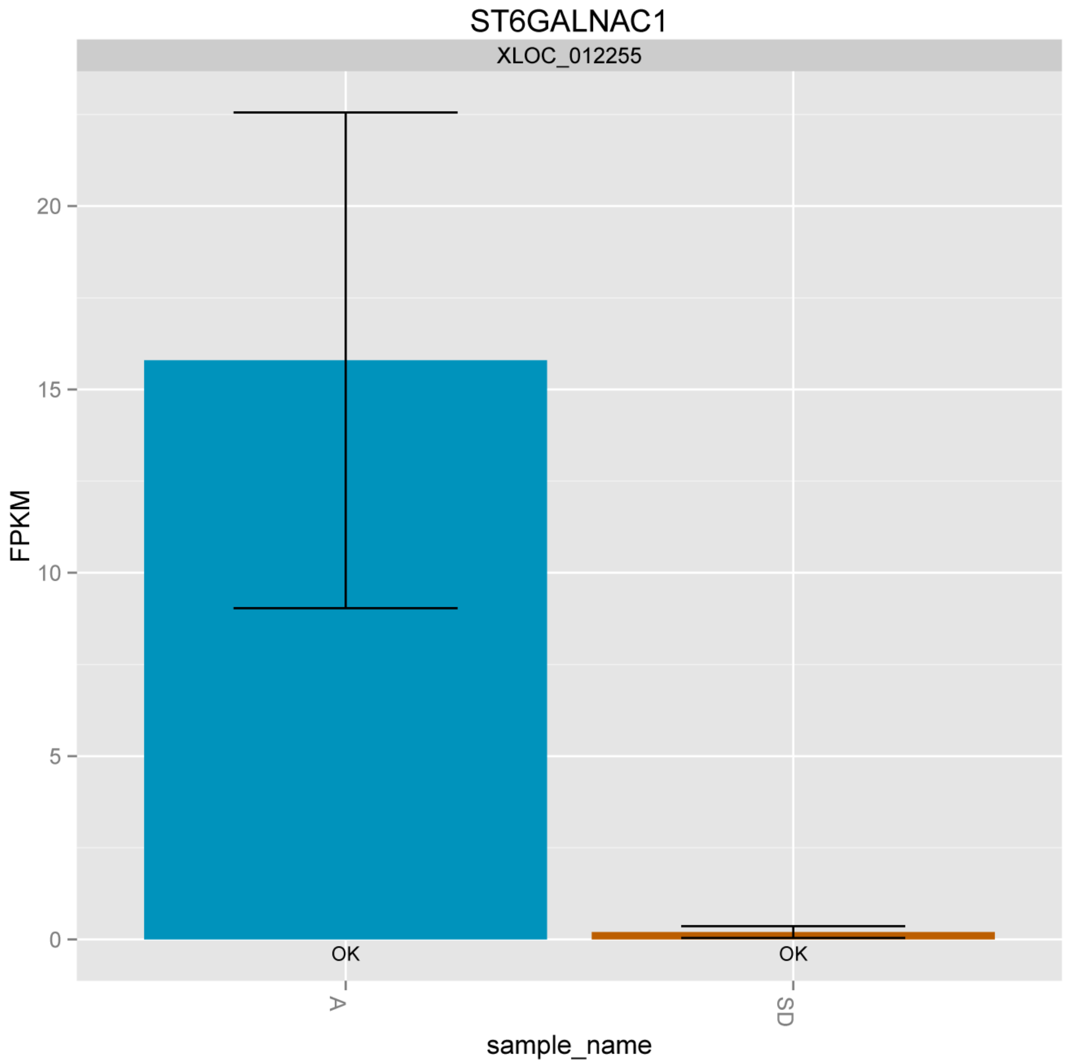

Supplementary Figure 4

ST6GalNAc1 exon 2 expression in RNA-seq analysis.

A

i)

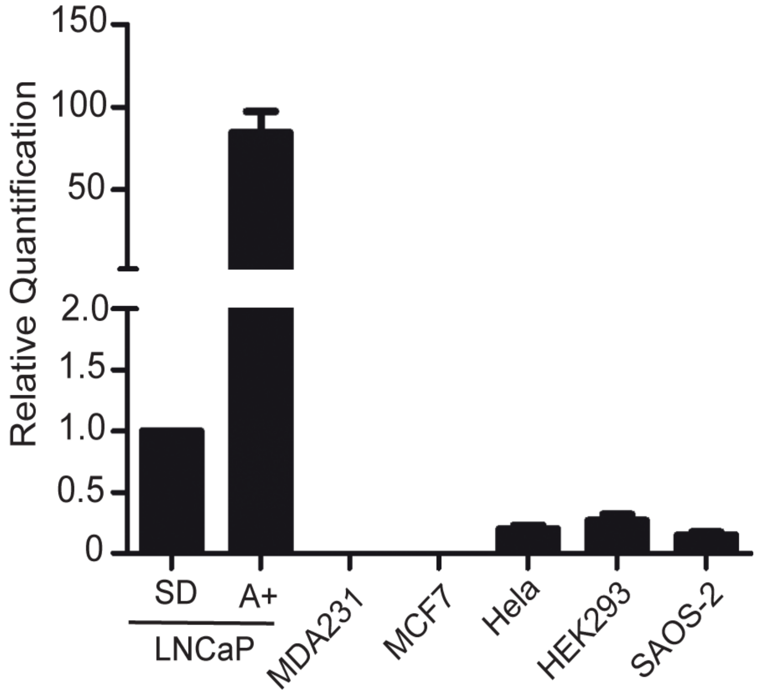

150
100
50
2.0
1.5
1.0
0.5
0
Relative Quantification
SD
A+
LNCaP
MDA231
MCF7
Hela
HEK293
SAOS-2

ii)

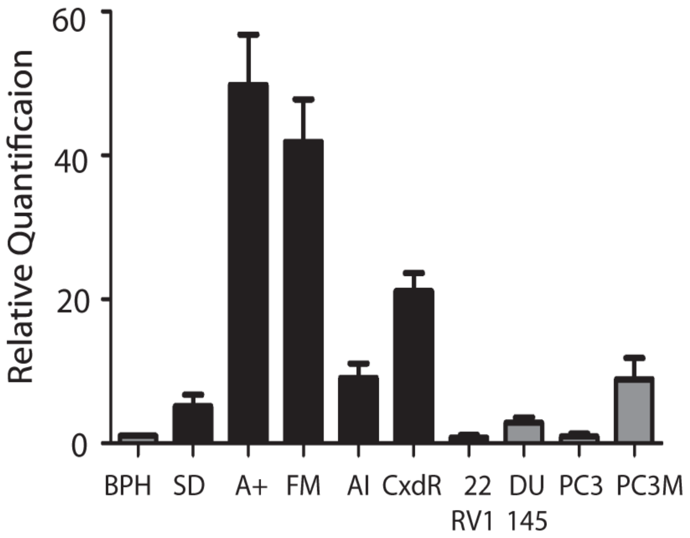

60
40
20
0
Relative Quantificaion
BPH
SD
A+
FM
AI
CxdR
22 RV1
DU 145
PC3
PC3M

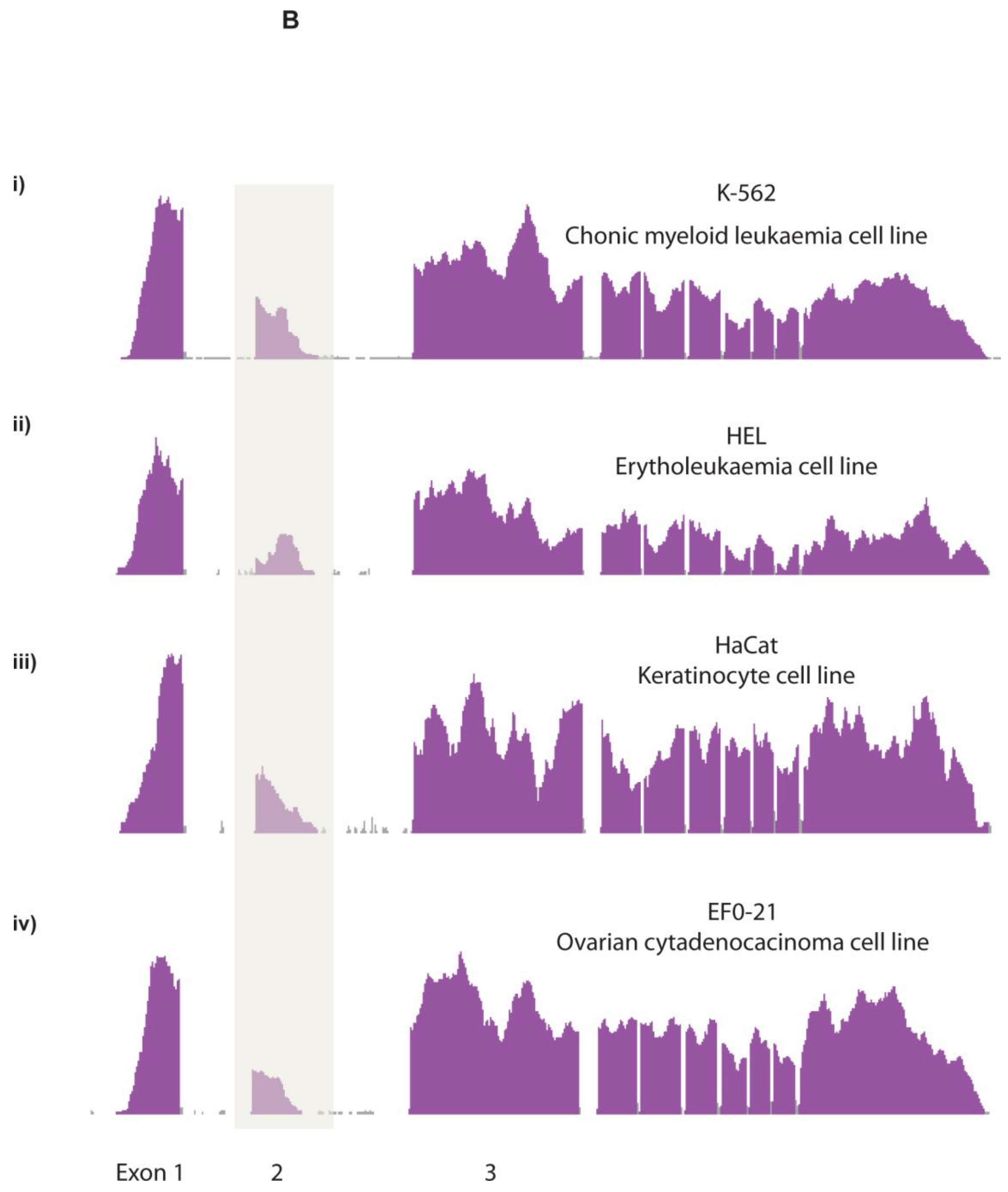

B
i)
K-562
Chonic myeloid leukaemia cell line
ii)
HEL
Erytholeukaemia cell line
iii)
HaCat
Keratinocyte cell line
iv)
EF0-21
Ovarian cytadenocacinoma cell line
Exon 1
2
3

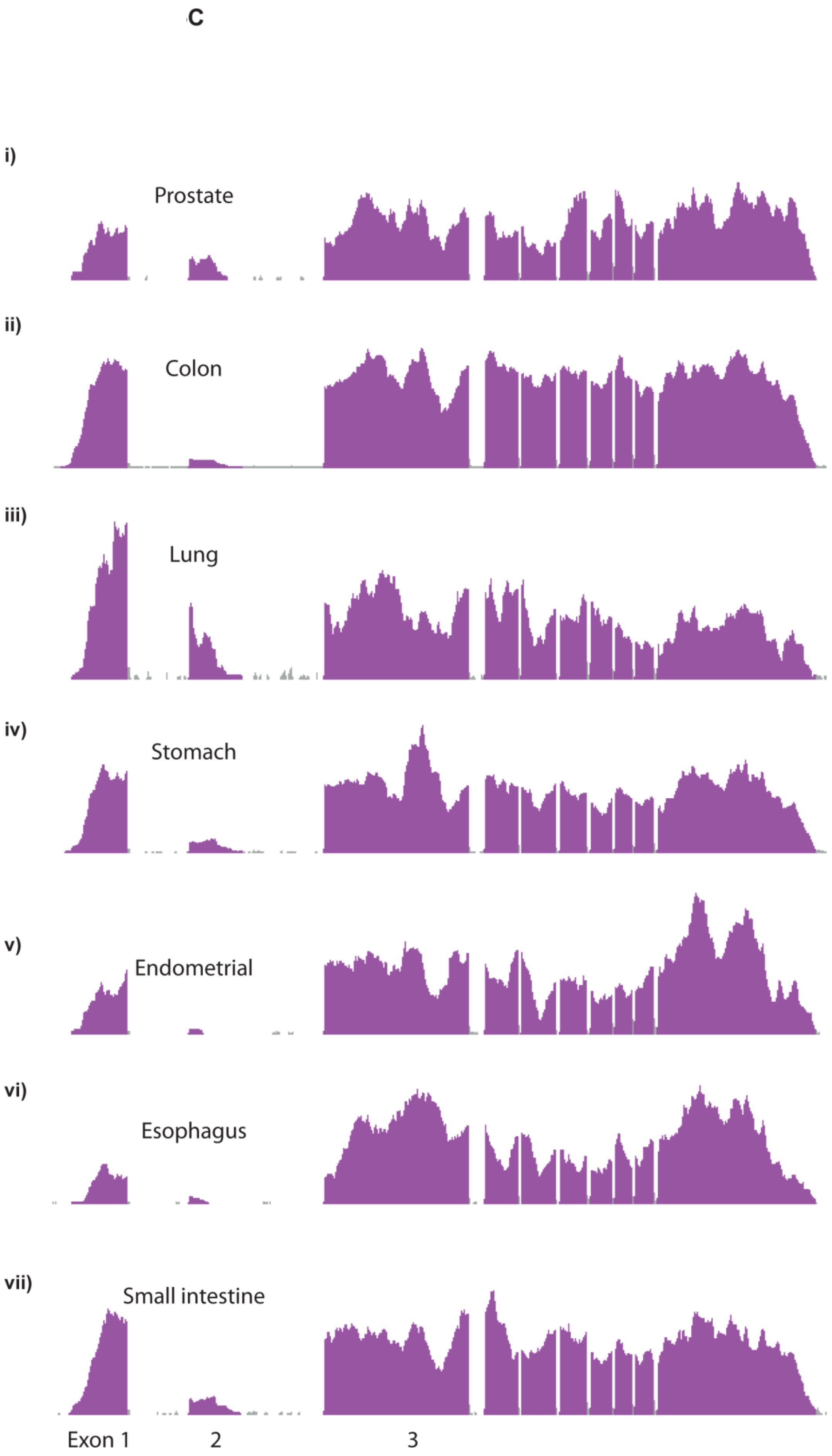
C
i)
Prostate
ii)
Colon
iii)
Lung
iv)
Stomach
v)
Endometrial
vi)
Esophagus
vii)
Small intestine
Exon 1
2
3

Supplementary Figure 5

(**A**) (i) Expression of ST6GalNac1 is a panel of non-PCa cell lines. The cell lines used were androgen responsive LNCaP PCa cells grown in the absence (steroid deplete, SD) or presence of androgens (A+), two breast cancer cell lines: MDA-231 (ATCC®HTB-26™) and MCF7 (ATCC® HTB-22™), which have been previously shown to lack ST6GalNAc1 (Julian et al 2001), Hela cells (ATCC® CCL-2™) , HEK293 cells (ATCC® CRL-1573™) and SAOS-2 (ATCC® HTB-85™). (ii) Real-time PCR using primers specific to exon 2 detects *ST6GalNAc1-short* in a panel of PCa cell lines. Samples were normalised to a benign prostate hyperplasia (BPH) cell line. Exon 2 was detected in LNCaP cells grown in steroid deplete (SD), +androgens (A+) and full media (FM), as well as in our androgen insensitive LNCaP (AI) and Casodex resistance LNCaP (CxdR) cell lines. For details of cell lines used see Materials and Methods.

(**B**) ST6GalNac1 exon 2 is detected in RNA-Seq reads from non-Pca cell lines. Analysis of publically available RNA-Seq reads from 44 cell lines [1] detected *ST6GalNac1* in 4 non-PCa cell lines (>5 FPKM was used as the cut off). (**C**) *ST6GalNac1* exon 2 is detected in a range of cancer tissues by RNA-Sequencing. Analysis of publically available RNA-Seq reads from 36 tissues [1] shows that *ST6GalNac1* mRNA and specifically exon 2 are detected in prostate and a range of additional tissues, particularly in lung and tissues from the digestive tract.

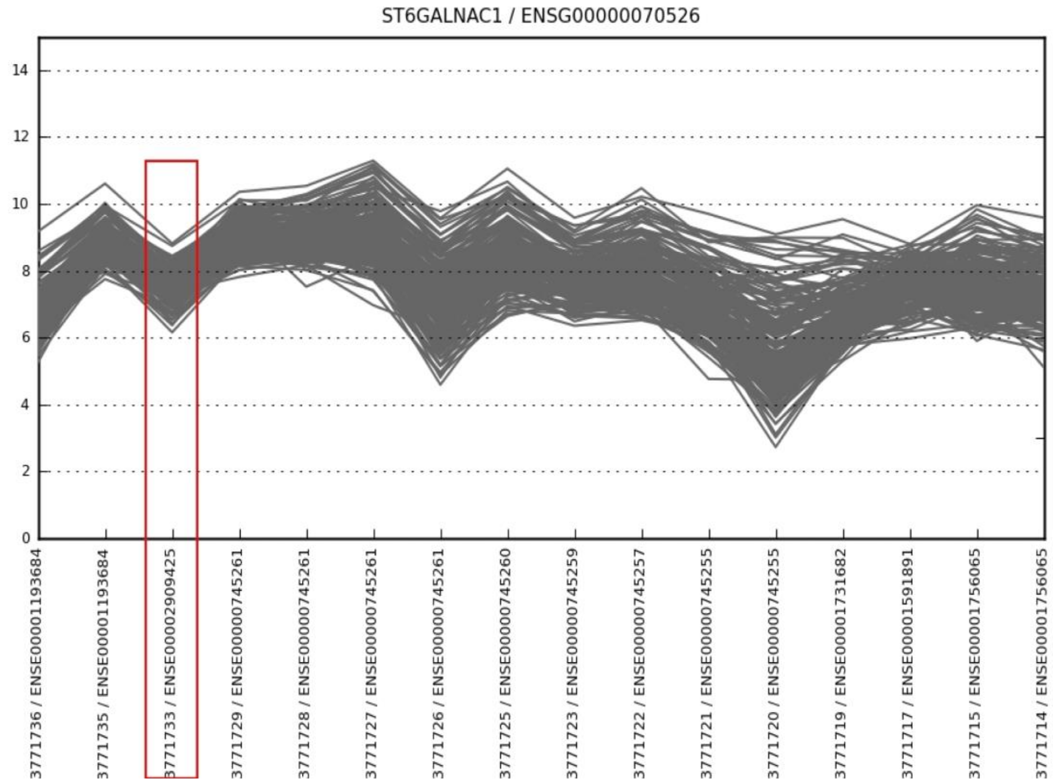

Supplementary Figure 6

Analysis of *ST6GalNAc1* in 185 PCas from the Taylor et al [2] dataset show that exon 2 is detected at similar levels to other exons, suggesting that *ST6GalNAc1-short* is the dominant isoform in these samples.

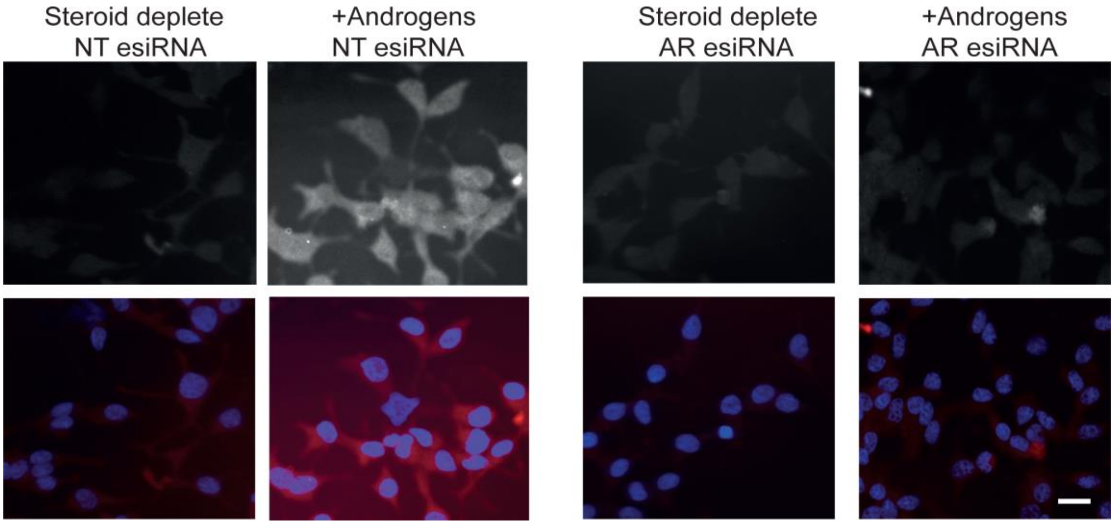

Supplementary Figure 7

Induction of sTn by androgens is inhibited by esiRNA mediated depletion of the androgen receptor (AR). Cells were pre-treated with either control or AR targeting esiRNA and then incubated in the presence (A+) or absence (SD) of 10nM R1881 (androgens) for 72 hours. Bar is 10 μm.

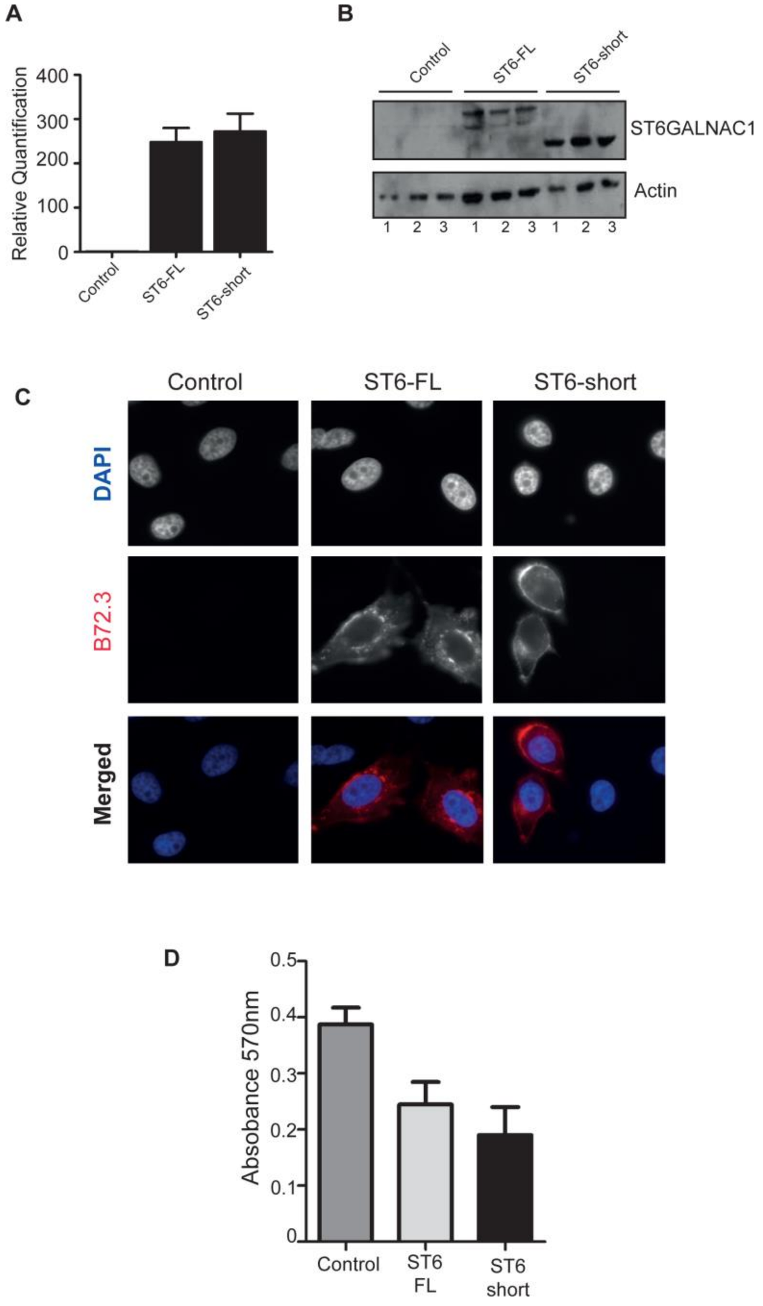

Supplementary Figure 8: Inducible over-expression of ST6GalNAc1 in PC3 cells induces expression of the sTn-antigen and reduces cell adhesion.

PC3 cells were induced to express ST6GalNAc1 full length or ST6GalNAc1-short by treatment with tetracycline for 72 hours (cells over-expressing empty vector were used as a control). Induction of *ST6GalNAc1* was confirmed at the RNA level by real-time PCR (A) and at the protein level by western blot (B). Expression of both

isoforms of ST6GalNAc1 induced expression of the sTn-antigen as detected by immunofluorescence (C) and reduced cell adhesion to uncoated plates (D).

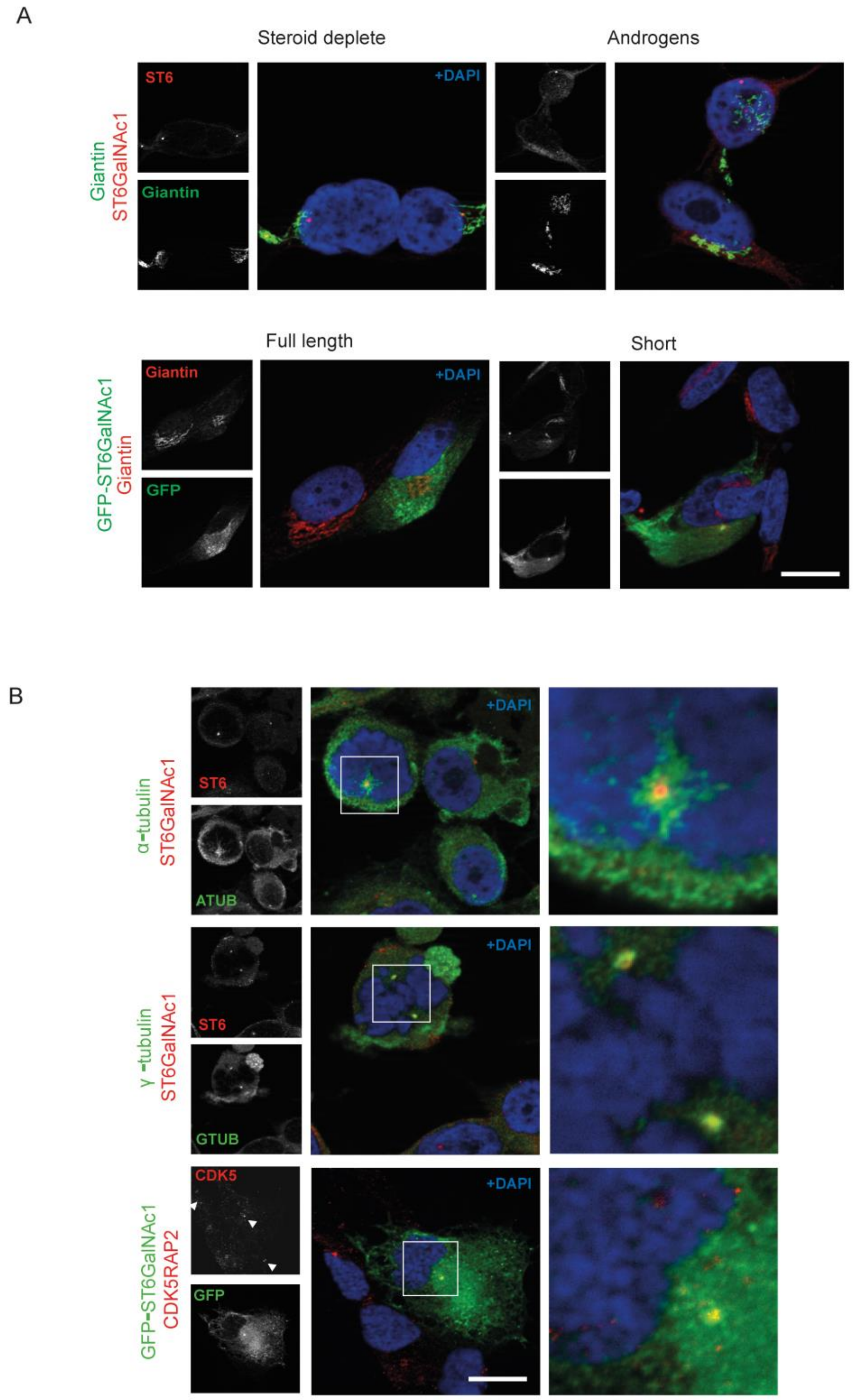

Supplementary Figure 9: Sub-cellular localisation of endogenous and GFP tagged ST6GalNAc1. (A) Analysis of ST6GalNAc1-short in LNCaP cells by immunofluorescence indicates a clear increase in expression in cells treated 10 nM

synthetic androgen analogue methyltrienolone (R1881) (androgens, A+) for 24 hours relative to cells grown in media supplemented with 10% charcoal dextran stripped FBS (steroid deplete media, SD) (A, upper panels). Co-localisation with golgi specific giantin indicates that in LNCaP cells ST6GalNAc1-short is localised throughout the cytoplasm rather than specifically to the golgi. (B) Co-localisation with α-tubulin and γ-tubulin reveals that in LNCaP cells endogenous *ST6GalNAc1-short* is specifically localised to the centrosome during mitosis (B, upper and middle panels). GFP-tagged ST6GalNAc1 was also transiently expressed in normal human dermal fibroblasts and co-stained with the centrosome marker CDK5RAP2 in non-dividing cells. ST6GalNAc1-short shows a clear localisation to the centrosome (B, lower panels). We did not find any evidence of centrosomal localisation for the full length ST6GalNAc1 isoform. Control of glycosylation via the sub-cellular localization of glycosyltransferases is a critical mechanism driving invasiveness of cancer cells [3]. ST6GalNAc1 has been previously shown to localise throughout the golgi apparatus [4]. The results presented here indicate that both full-length ST6GalNAc1 and ST6GalNAc1-short have a cytoplasmic sub-cellular localisation in LNCaP cells, suggesting potential aberrant glycosylation. Interestingly, our data also suggests that in addition to localising throughout the cytoplasm *ST6GalNAc1*-short, but not full length ST6GalNAc1, has a discrete centrosome-specific localisation. Bar is 10µm.

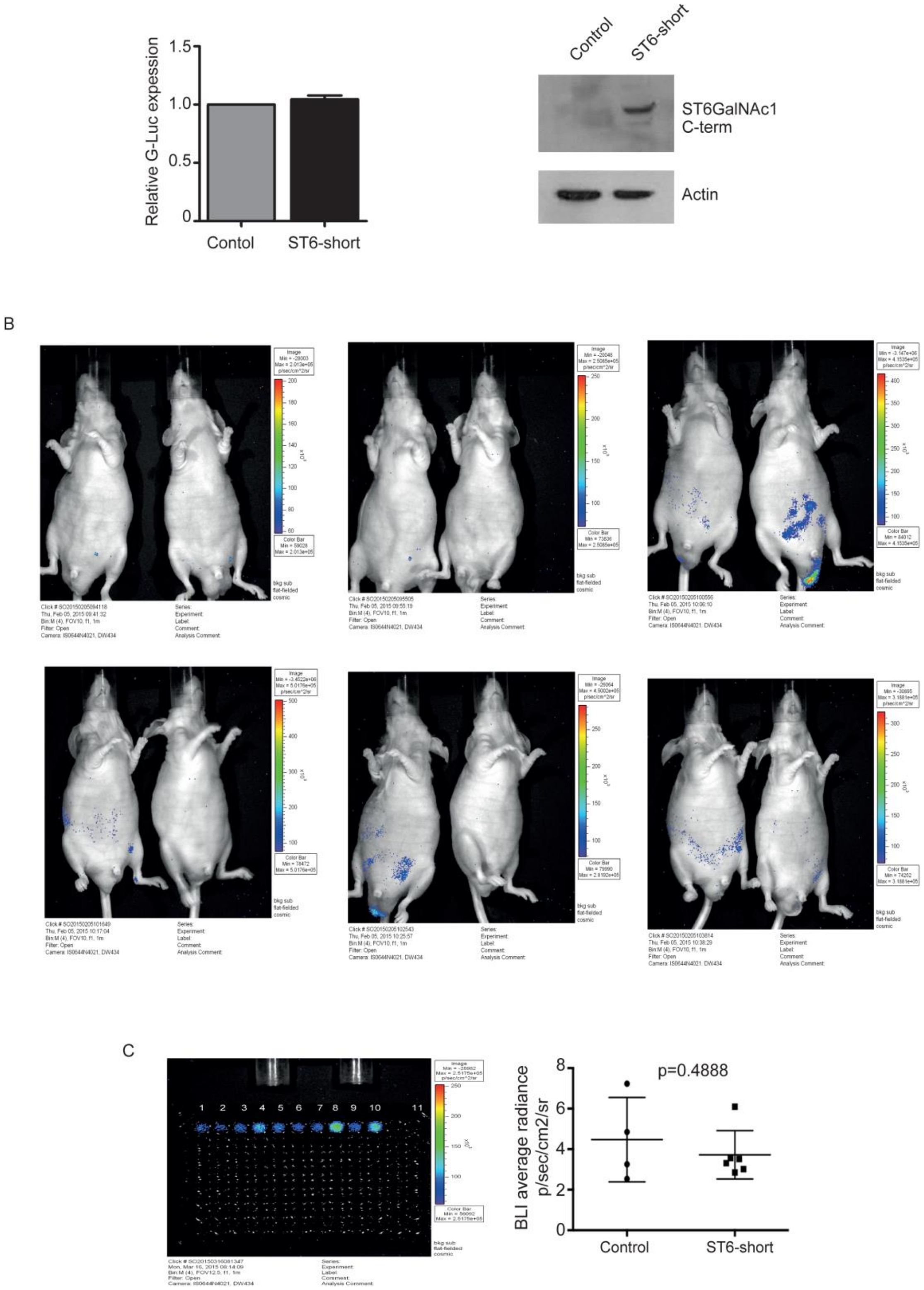

Supplementary Figure 10: In vivo metastasis assay

(A) DU145 stable cell lines overexpressing either control empty vector or ST6GalNAc1-short were transfected with Gaussia Luciferase. Relative Gaussia Luciferase activity was then determined and normalised to cell number. Colonies with equal levels were chosen for in vivo experiments. (B) Example of in vivo imaging of whole mice following coelenterazine injections intraperitoneally. In each picture on the left side is the mouse injected with ST6GalNAc1-short overexpressing cells and on the right side mouse injected with empty-vector control. Either no signal or weak signal above background was observed through-out the experiment, consistent with disseminated cells and not established metastatic foci. (C) G-Luc quantification in blood at 12 weeks. Monitoring urine and bloods showed that the mice had GLuc levels above background, however no difference was observed between the control and ST6GalNAc1-short overexpressing cells.

A. Representative images from cell migration assay

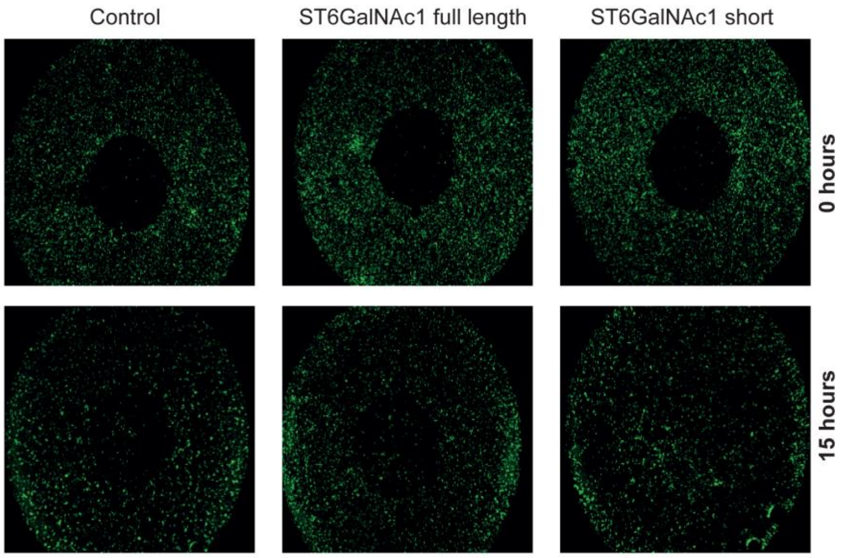

B. Adhesion assay: LNCaP cells transfected with control or ST6GalNAc1 esiRNA

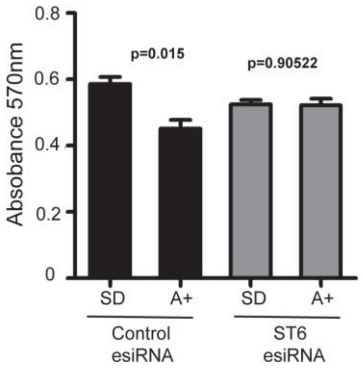

Supplementary Figure 11

(A) Migration assay images to supplement Figure 5B. (B) Adhesion assay: LNCaP cells transfected with control or *ST6GalNAc1* esiRNA. LNCaP cells grown in the presence (A+) of absence (SD) of 10nM R1881 were transfected with *ST6GalNAc1* esiRNA. After 72 hours cell adhesion to uncoated plates was determined. For control

cells there was a significant reduction in cell adhesion following androgen treatment (p=0.015), which was not seen for cells depleted of ST6GalNAc1 protein (p=0.905).

Supplementary References

1. Uhlen, M., et al., *Proteomics. Tissue-based map of the human proteome.* Science, 2015. **347**(6220): p. 1260419.
2. Taylor, B.S., et al., *Integrative genomic profiling of human prostate cancer.* Cancer Cell, 2010. **18**(1): p. 11-22.
3. Gill, D.J., et al., *Initiation of GalNAc-type O-glycosylation in the endoplasmic reticulum promotes cancer cell invasiveness.* Proc Natl Acad Sci U S A, 2013. **110**(34): p. E3152-61.
4. Sewell, R., et al., *The ST6GalNAc-I sialyltransferase localizes throughout the Golgi and is responsible for the synthesis of the tumor-associated sialyl-Tn O-glycan in human breast cancer.* J Biol Chem, 2006. **281**(6): p. 3586-94.
