# Supplementary material for: The androgen receptor controls expression of the cancer-associated sTn antigen and cell adhesion through induction of ST6GalNAc1 in prostate cancer
Source: Oncotarget. 2015 Oct 7;6(33):34358–74. doi: 10.18632/oncotarget.6024 (PMC4741458; doi:10.18632/oncotarget.6024)
Supplement: Supplementary file 2 [file oncotarget-06-34358-s002.pdf]

Supplementary Table 1

Primer sequences

Target	Forward	Reverse
GAPDH	AAC AGC GAC ACC CAT CCT C	CAT ACC AGG AAA TGA GCT TGA CAA
β-tubulin	CTTCGGCCAGATCTTCAGAC	AGAGAGTGGGTCAGCTGGAA
Actin	CATCGAGCACGGCATCGTCA	TAGCACAGCCTGGATAGCAAC
CAMKK2	TGAAGACCAGGCCCGTTTCTACTT	TGGAAGGTTTGATGTCACGGTGGA
ABCC4	TTCTTCTGGTGGCTCAATCC	TTCTGTGCGTCATTCTCAGC
ST6GalNAc1	AGGCACAGACCCCAGGAAG	TGAAGCCATAAGCACTCACC
ST6GalNAc2	CACCCCTCCAAAGTGTATCC	AAGCCCAGATTCCAGTAGGA
ST6GalNAc3	GCTGGTTGTGCGTCTTGTAA	TCTCATTTCCCACCTTCTGG
ST6GalNAc4	CAGTCGGGCTCCTTCCTC	CTGCTCGTGTGCCAGGTA
ST6GalNAc5	AAGCAGGAGACTGGCAAAGA	CATCAGGTCCAAAAGGTTCA
ST6GalNAc6	GGCAAGGACAGGGAGAAGTC	GGGCTCGTAGTAGTGGTAGGG
ST6GalNAc1 ex1-2	GGTCCTGCCTGTGGAGATG	CCTGGAGCCCTTGATACC
ST6GalNAc1 ex2-3	GCTGACCCTTCGCTATTGAG	GTTGTCCTCCTTGCCCTTGT
ST6GalNAc1 exon 1-3 (Figure 3C)	CTTCTTTCTCTTCGCCTTGC	TCTTTTTCTGGGCTCTTCCA
E-cadherin	TTCCCAACTCCTCTCCTG	AAACCTTGCCTTCTTTGTC
N-cadherin	CTCCTATGAGTGGAACAGGAACG	TTGGATCAATGTCATAATCAAGTGCTGTA
Vimentin	TGTCCAAATCGATGTGGATGTTTC	TTGTACCATTCTTCTGCCTCCTG
Snail	GAGGCGGTGGCAGACTAG	GACACATCGGTCAGACCAG
Slug	CATGCCTGTCATACCACAAC	GGTGTCAGATGGAGGAGGG
β-microglobulin	CCAGCAGAGAATGGAAAGTC	GATGCTGCTTACATGTCTCG
β-catenin	GGCTACTCAAGCTGATTTGATGGA	AAGACTGTTGCTGCCAGTGACTA
Fibronectin	TGGGTGACACTTATGAGCGTC	TCCCACGTTTCTCCGACCA
KLK3	TGACCAAGTTCATGCTGTGT	GTCATTTCCAAGGTTCCAAG
ChIP-qPCR IM_126-1	CTGAGCTACTGTGGACAATC	GAACCTGAGAACCAGGAAAC
ChIP-qPCR IM_126-2	GGCATTTCTCCTAACGTACAG	CCTTGAGGAAGGTGGTTTAAT
ChIP-qPCR IM_9298-1	CAGGGCTTTGTTTGTGGTAG	CGTGCTGGTCATTGTTGTAA
ChIP-qPCR IM_9298-2	CTACCCTGTGGAATGCTAAG	CACAGGTTGACCTTGTAAGT
ChIP-qPCR IM_9298-3	CCTAATATTGATCATTGCTCGC	TAGCCACTTGAGAATGAATCAC
ChIP-qPCR IM_9298-4	GCATCTCACTCTTCTGGTTT	CGACACTGTGATGTGGTTTA
ChIP-qPCR IM_9298-5	CTCATGGTTGATGTCTCTCTTT	GCCTCCTGTCTATTCTTGATATT
ChIP-qPCR Neg2	CCTTTCTTGGCAACCATTTC	AGCAGTGGTGTAATATGATCTC
ChIP-qPCR Neg1	CAGAGGGCTTCTGGTGAAAC	TTGACAATGTCTTGCCTTGG
