# Supplementary material for: The androgen receptor controls expression of the cancer-associated sTn antigen and cell adhesion through induction of ST6GalNAc1 in prostate cancer
Source: Oncotarget. 2015 Oct 7;6(33):34358–74. doi: 10.18632/oncotarget.6024 (PMC4741458; doi:10.18632/oncotarget.6024)
Supplement: Supplementary file 6 [file oncotarget-06-34358-s006.pdf]

Genes identified as upregulated by RNA-Seq which overlap with Massie et al.
27 genes were identified as novel androgen-regulated genes and validated by real-time PCR

Gene	Distance to AR binding site (kb)	RNASeq fold change (log2)	RT-qPCR fold change	Regulated by androgens	Reference
ABCC4	26.27	-2.87035	-	Y	[1]
ACSL3	43.46	-4.08103	-	Y	[2]
ALDH1A3	-	-2.11804	-	Y	[3]
ATAD2	11.91	-3.10765	-	Y	[4]
BMPR1A	12.39	-1.88759	-	Y	[5]
C1ORF21	6.13	-2.43628	-	Y	[6]
CAMKK2	2.05	-2.48781	-	Y	[7]
CCDC141	17.02	-9.24299	5.361	-	-
CECR6	3.27	-4.73026	2.01	-	-
CENPN	26.55	-4.45106	-	-	-
CXCR4	18.57	-3.09835	-	Y	[8]
DBI	1.08	-3.46779	-	Y	[9]
ELK4	19.56	-3.23858	-	Y	[10]
FZD5	12.19	-3.18877	-	Y	[11]
HERC3	39.58	-2.4694	5.62	-	-
HS3ST1	12.08	-2.77995	6.530	-	-
IGF1R	13.28	-4.34758	-	Y	[12]
INSIG1	3.33	-3.00032	-	Y	[13]
KCNN2	2.22	-2.1997	4.270	-	-
LAMA1	25.16	-4.90533	6.74	-	-
LPIN1	1.32	-1.63235	3.740	Y	[14]
LRIG1	0.41	-2.40902	-	Y	[15]
LRRFIP2	46.22	-2.03091	5.070	-	-
MAK	32.09	-2.68438	-	Y	[16]
MAP7D1	24.15	-2.20145	5.870	-	-
MBOAT2	7.29	-3.06514	8.82	-	-
MPHOSPH9	12.12	-2.17375	5.7	-	-
NCAPD3	7.16	-5.88544	-	Y	[17]
NPPC	4.50	-6.02878	4.854	-	-
PAK1IP1	13.12	-3.37467	11.380	-	-
PCTP	96.92	-2.00041	5.580	-	-
PGC	14.32	-5.77086	-	Y	[18]
PPAP2A	30.24	-2.60712	7.300	-	-
PPAPDC2	13.73	-1.83803	11.319	-	-
PPF1BP2	2.32	-1.6414	-	-	-
PTPN21	5.25	-2.78769	12.090	-	-
RAB4A	9.78	-1.73694	3.500	-	-
SASH1	19.16	-2.06417	4.180	-	-
SHROOM3	7.75	-1.6095	3.020	-	-
SLC26A3	2.46	-6.95308	157.510	-	-
SLC45A3	67.54	-1.8775	-	Y	[19]
SOCS2	23.45	-3.17726	-	Y	[20]
SPHAR	1.92	-1.73694	-	-	-
ST6GALNAC1	2.64	-6.29347	68.94	-	-
STK39	11.91	-2.87335	-	-	-
TBC1D8	32.88	-2.35501	-	Y	[21]
TNFAIP3	2.70	-2.75714	-	Y	[22]
TNFRSF10B	1.60	-1.99227	-	-	-
UBE2G1	6.42	-1.65387	3.420	-	-
WIPI1	48.17	-4.17212	16.750	-	-
ZCCHC6	22.42	-1.70302	3.010	-	-

1. Ho, L.L., et al., *Androgen regulation of multidrug resistance-associated protein 4 (MRP4/ABCC4) in prostate cancer.* Prostate, 2008. **68**(13): p. 1421-9.
2. Hendriksen, P.J., et al., *Evolution of the androgen receptor pathway during progression of prostate cancer.* Cancer Res, 2006. **66**(10): p. 5012-20.
3. Trasino, S.E., E.H. Harrison, and T.T. Wang, *Androgen regulation of aldehyde dehydrogenase 1A3 (ALDH1A3) in the androgen-responsive human prostate cancer cell line LNCaP.* Exp Biol Med (Maywood), 2007. **232**(6): p. 762-71.
4. Zou, J.X., et al., *Androgen-induced coactivator ANCCA mediates specific androgen receptor signaling in prostate cancer.* Cancer Res, 2009. **69**(8): p. 3339-46.
5. Ide, H., et al., *Cloning of human bone morphogenetic protein type IB receptor (BMPR-IB) and its expression in prostate cancer in comparison with other BMPRs.* Oncogene, 1997. **14**(11): p. 1377-82.
6. DePrimo, S.E., et al., *Transcriptional programs activated by exposure of human prostate cancer cells to androgen.* Genome Biol, 2002. **3**(7): p. RESEARCH0032.
7. Massie, C.E., et al., *The androgen receptor fuels prostate cancer by regulating central metabolism and biosynthesis.* EMBO J, 2011. **30**(13): p. 2719-33.
8. Akashi, T., et al., *Androgen receptor negatively influences the expression of chemokine receptors (CXCR4, CCR1) and ligand-mediated migration in prostate cancer DU-145.* Oncol Rep, 2006. **16**(4): p. 831-6.
9. Swinnen, J.V., et al., *Androgen regulation of the messenger RNA encoding diazepam-binding inhibitor/acyl-CoA-binding protein in the human prostatic adenocarcinoma cell line LNCaP.* Mol Cell Endocrinol, 1994. **104**(2): p. 153-62.
10. Makkonen, H., et al., *Identification of ETS-like transcription factor 4 as a novel androgen receptor target in prostate cancer cells.* Oncogene, 2008. **27**(36): p. 4865-76.
11. Thiele, S., et al., *Expression profile of WNT molecules in prostate cancer and its regulation by aminobisphosphonates.* J Cell Biochem, 2011. **112**(6): p. 1593-600.
12. Krueckl, S.L., et al., *Increased insulin-like growth factor I receptor expression and signaling are components of androgen-independent progression in a lineage-derived prostate cancer progression model.* Cancer Res, 2004. **64**(23): p. 8620-9.
13. Heemers, H.V., G. Verhoeven, and J.V. Swinnen, *Androgen activation of the sterol regulatory element-binding protein pathway: Current insights.* Mol Endocrinol, 2006. **20**(10): p. 2265-77.
14. Haren, M.T., et al., *Testosterone modulates gene expression pathways regulating nutrient accumulation, glucose metabolism and protein turnover in mouse skeletal muscle.* Int J Androl, 2011. **34**(1): p. 55-68.
15. Thomasson, M., et al., *LRIG1 and the liar paradox in prostate cancer: a study of the expression and clinical significance of LRIG1 in prostate cancer.* Int J Cancer, 2011. **128**(12): p. 2843-52.
16. Ma, A.H., et al., *Male germ cell-associated kinase, a male-specific kinase regulated by androgen, is a coactivator of androgen receptor in prostate cancer cells.* Cancer Res, 2006. **66**(17): p. 8439-47.
17. Lapointe, J., et al., *hCAP-D3 expression marks a prostate cancer subtype with favorable clinical behavior and androgen signaling signature.* Am J Surg Pathol, 2008. **32**(2): p. 205-9.
18. Shiota, M., et al., *Peroxisome proliferator-activated receptor gamma coactivator-1alpha interacts with the androgen receptor (AR) and promotes prostate cancer cell growth by activating the AR.* Mol Endocrinol, 2010. **24**(1): p. 114-27.
19. Xu, J., et al., *Identification and characterization of prostein, a novel prostate-specific protein.* Cancer Res, 2001. **61**(4): p. 1563-8.
20. Iglesias-Gato, D., et al., *SOCS2 mediates the cross talk between androgen and growth hormone signaling in prostate cancer.* Carcinogenesis, 2014. **35**(1): p. 24-33.
21. Lehmusvaara, S., et al., *Chemical castration and anti-androgens induce differential gene expression in prostate cancer.* J Pathol, 2012. **227**(3): p. 336-45.

22. Golovko, O., N. Nazarova, and P. Tuohimaa, *A20 gene expression is regulated by TNF, Vitamin D and androgen in prostate cancer cells.* J Steroid Biochem Mol Biol, 2005. **94**(1-3): p. 197-202.
