# Supplementary material for: The androgen receptor controls expression of the cancer-associated sTn antigen and cell adhesion through induction of ST6GalNAc1 in prostate cancer
Source: Oncotarget. 2015 Oct 7;6(33):34358–74. doi: 10.18632/oncotarget.6024 (PMC4741458; doi:10.18632/oncotarget.6024)
Supplement: Supplementary file 7 [file oncotarget-06-34358-s007.pdf]

**Genes identified as downregulated by RNA-Seq which overlap with Massie et al.**
33 genes were identified as novel androgen-regulated genes and validated by real-time PCR

| Gene | Distance to AR binding site (kb) | RNASeq fold change (log2) | RT-qPCR fold change | Regulated by androgens | Reference | Fold change Grasso *et al* |
|---|---|---|---|---|---|---|
| ADCY1 | 23.49 | -318,628 | 0.22 | Y | [1] | 1.232 |
| AHNAK2 | 8.59 | -364,918 | 0.09 | - | - | -2.446 |
| BTG2 | 40.42 | -216,509 | - | Y | [2] | -1.167 |
| C5orf30 | 30.79 | -233,042 | 0.11 | - | - | 1.569 |
| CA12 | 11.03 | -199,921 | - | - | - | -1.693 |
| CAMK2N1 | 5.7 | -476,043 | - | Y | [3] | -1.279 |
| CCNG2 | 3.95 | -22,341 | 0.1 | - | - | 1.026 |
| CDH26 | 42.43 | -350,374 | 0.1 | - | - | -1.527 |
| COLEC12 | 26.60 | -26,723 | 0.1 | - | - | - |
| DDC | 11.94 | -351,923 | - | Y | [4] | -1.260 |
| DSEL | 32.89 | -237,839 | 0.08 | - | - | -1.429 |
| ENC1 | 8.76 | -237,885 | 0.08 | - | - | 1.558 |
| GRB10 | 2.93 | -281,377 | 0.08 | Y | [5] | -1.316 |
| KCNS3 | 36.96 | -322,383 | - | - | - | 1.385 |
| LRRN1 | 11.26 | -448,499 | - | - | - | -1.083 |
| MAN1A1 | 1.63 | -38,368 | 0.03 | - | - | -1.117 |
| MANEA | 31.05 | -324,525 | 0.15 | Y | [6] | 1.575 |
| MAP2K6 | 5.68 | -305,452 | 0.15 | - | - | 1.350 |
| MMP16 | 0.87 | -42,541 | 0.02 | Y | [7] | -1.756 |
| MYC | 43.92 | -192,062 | - | - | - | -1.079 |
| OPRK1 | 5.26 | -502,389 | 0.03 | Y | [8] | -1.659 |
| OSR2 | 1.21 | -300,426 | 0.07 | - | - | -1.760 |
| PBX1 | 25.18 | -285,457 | - | Y | [9] | -1.863 |
| PER3 | 28.62 | -232,226 | 0.13 | - | - | -1.918 |
| PKIB | 1.3 | -298,603 | - | Y | [10] | 2.224 |
| SEMA6D | 1.05 | -396,007 | 0.05 | - | - | -2.259 |
| SLC44A1 | 30.77 | -227,105 | 0.13 | - | - | -1.033 |
| SPRY1 | 1.95 | -28,233 | - | Y | [11] | -1.123 |
| SPTLC3 | 26.18 | -293,737 | 0.06 | - | - | -1.636 |
| SRGAP3 | 29.89 | -254,123 | 0.16 | - | - | -1.384 |
| SYTL2 | 36.06 | -483,007 | - | Y | [12] | 1.271 |
| TARBP1 | 5.05 | -209,915 | 0.12 | - | - | 1.421 |
| TGM3 | 11.37 | -569,763 | - | Y | [12] | 3.489 |
| TLN2 | 15.43 | -262,683 | 0.08 | - | - | 1.100 |
| TLR3 | 7.1 | -33,068 | 0.03 | Y | [13] | -1.492 |
| TMEM144 | 39.76 | -257,624 | 0.16 | - | - | 1.400 |
| TNFSF15 | 7.3 | -268,553 | 0.11 | - | - | -1.497 |
| TRIB1 | 0.78 | -255,395 | 0.24 | Y | [1] | 1.996 |
| ZIC2 | 31.74 | -418,594 | 0.03 | - | - | 2.707 |


1. Munkley, J., et al., *Androgen-regulation of the protein tyrosine phosphatase PTPRR activates ERK1/2 signalling in prostate cancer cells.* BMC Cancer, 2015. **15**: p. 9.
2. Jalava, S.E., et al., *Androgen-regulated miR-32 targets BTG2 and is overexpressed in castration-resistant prostate cancer.* Oncogene, 2012. **31**(41): p. 4460-4471.

3. Romanuik, T.L., et al., *Novel biomarkers for prostate cancer including noncoding transcripts.* Am J Pathol, 2009. **175**(6): p. 2264-76.
4. Wafa, L.A., et al., *Carbidopa abrogates L-dopa decarboxylase coactivation of the androgen receptor and delays prostate tumor progression.* Int J Cancer, 2012. **130**(12): p. 2835-44.
5. Haren, M.T., et al., *Testosterone modulates gene expression pathways regulating nutrient accumulation, glucose metabolism and protein turnover in mouse skeletal muscle.* Int J Androl, 2011. **34**(1): p. 55-68.
6. Romanuik, T.L., et al., *Identification of novel androgen-responsive genes by sequencing of LongSAGE libraries.* BMC Genomics, 2009. **10**: p. 476.
7. Delassus, G.S., et al., *Many new down- and up-regulatory signaling pathways, from known cancer progression suppressors to matrix metalloproteinases, differ widely in cells of various cancers.* J Cell Physiol, 2010. **224**(2): p. 549-58.
8. Romanuik, T.L., et al., *LNCaP Atlas: gene expression associated with in vivo progression to castration-recurrent prostate cancer.* BMC Med Genomics, 2010. **3**: p. 43.
9. Alshbib, A. *PBX expression and regulation in human prostate*. [Masters Thesis] 2009 2010; Available from: https://www.duo.uio.no/handle/10852/29533?show=full.
10. Chung, S., et al., *Overexpressing PKIB in prostate cancer promotes its aggressiveness by linking between PKA and Akt pathways.* Oncogene, 2009. **28**(32): p. 2849-59.
11. Terada, N., et al., *Correlation of Sprouty1 and Jagged1 with aggressive prostate cancer cells with different sensitivities to androgen deprivation.* J Cell Biochem, 2014. **115**(9): p. 1505-15.
12. Margiotti, K., et al., *Androgen-regulated genes differentially modulated by the androgen receptor coactivator L-dopa decarboxylase in human prostate cancer cells.* Mol Cancer, 2007. **6**: p. 38.
13. Gambara, G., et al., *TLR3 engagement induces IRF-3-dependent apoptosis in androgen-sensitive prostate cancer cells and inhibits tumour growth in vivo.* J Cell Mol Med, 2015. **19**(2): p. 327-39.
